# Bacterial-derived exopolysaccharides enhance antifungal drug tolerance in a cross-kingdom oral biofilm

**DOI:** 10.1038/s41396-018-0113-1

**Published:** 2018-04-18

**Authors:** Dongyeop Kim, Yuan Liu, Raphael I. Benhamou, Hiram Sanchez, Áurea Simón-Soro, Yong Li, Geelsu Hwang, Micha Fridman, David R. Andes, Hyun Koo

**Affiliations:** 1 Biofilm Research Laboratory, Department of Orthodontics and Divisions of Pediatric Dentistry & Community Oral Health, School of Dental Medicine, University of Pennsylvania, Philadelphia, PA USA; 2 School of Chemistry, Raymond & Beverly Sackler Faculty of Exact Sciences, Tel Aviv University, Tel Aviv, Israel; 3 Departments of Medicine and Medical Microbiology and Immunology, University of Wisconsin, Madison, WI USA

## Abstract

Fungal–bacterial interactions generate unique biofilms that cause many infections in humans. *Candida albicans* interact with *Streptococcus mutans* in dental biofilms associated with severe childhood tooth-decay, a prevalent pediatric oral disease. Current modalities are ineffective and primarily based on antimicrobial monotherapies despite the polymicrobial nature of the infection. Here, we show that the combination of clinically used topical antifungal fluconazole with povidone iodine (PI) can completely suppress *C. albicans* carriage and mixed-biofilm formation without increasing bacterial killing activity *in vivo*. We unexpectedly found that the inclusion of PI enhanced fluconazole efficacy by potently disrupting the assembly of a protective bacterial exopolysaccharide (EPS) matrix through inhibition of α-glucan synthesis by *S. mutans* exoenzyme (GtfB) bound on the fungal surface. Further analyses revealed that the EPS produced *in situ* directly bind and sequester fluconazole, reducing uptake and intracellular transportation of the drug. Conversely, inhibition of GtfB activity by PI, enzymatic degradation of the α-glucan matrix or co-culturing with *gtfB*-defective *S. mutans* re-established antifungal susceptibility. Hence, topical antifungal has limitations in mixed oral biofilms due to enhanced *C. albicans* tolerance to fluconazole afforded by the shielding effect of bacterial-derived EPS. The data provide new insights for treatment of *C. albicans* in cross-kingdom biofilms, indicating that EPS inhibitors may be required for enhanced killing efficacy and optimal anti-biofilm activity.

## Introduction

Polymicrobial interactions, particularly involving fungi and bacteria, commonly occur in various sites of the human body, leading to pathogenic biofilms that are associated with many localized infections [1–3]. These cross-kingdom biofilms are structurally complex and challenging to eradicate, displaying enhanced tolerance to antimicrobials *in vitro* [4, 5]. Yet, most of the clinically used therapeutic approaches are monotherapies based on either antibacterial or antifungal agents despite the polymicrobial nature of disease-causing biofilms [6, 7]. Thus, enhanced understanding of the therapeutic implications of bacterial–fungal biofilms *in vivo* could help design improved antibiofilm strategies and overcome the limitations of current therapies.

*Candida albicans* is the most prevalent fungal pathogen causing oral and systemic infections [1, 3, 8, 9]. The ability of this organism to infect and cause diseases is associated with biofilm formation, often involving interactions with bacteria on mucosal surfaces [2, 3, 7, 10]. Intriguingly, *C. albicans* can also interact with *Streptococcus mutans* on hard tissue (tooth) surfaces to form mixed-kingdom biofilms associated with early childhood caries (ECC) (as reviewed in [11]). ECC is a severe form of tooth decay that affects underprivileged pre-school children exposed to sugar-rich diet and constitutes a major global public health problem [12]. The interactions between *C. albicans* and *S. mutans* dramatically modifies the biofilm environment by boosting the amounts of extracellular polysaccharides (EPS), which increases the bulk of the biofilm and the density of infection *in vivo*, enhancing the cariogenic potential of the biofilm [13–15].

The mixed biofilm contains an extensive extracellular matrix rich in insoluble α-glucan that is produced primarily by *S. mutans*-derived exoenzymes termed glucosyltransferases (Gtfs) using sucrose as a substrate [16]. The presence of *C. albicans* induces the *gtfB* expression in *S. mutans* and the secreted exoenzymes [Glucosyltransferase B (GtfB)] binds avidly to the fungal surface in active form, producing copious amounts of α-glucans *in situ* [13, 14]. The EPS produced on surrogate *Candida* surface enhance co-adhesion and promote mixed-biofilm development with *S. mutans* on tooth surfaces [13, 17]. Therefore, targeting both the bacterial and fungal cells may be required for effective elimination of this highly pathogenic oral biofilm, while the presence of elevated amounts of bacterially derived EPS surrounding the fungal cells could provide protection against antifungals. Here, we examined whether two clinically used topical oral antimicrobials, povidone iodine (PI) and fluconazole, can disrupt cross-kingdom biofilms.

PI has been used to reduce salivary levels of *S. mutans* in children affected by ECC although it is less effective against biofilm cells [18, 19]. Fluconazole is extensively used to prevent and treat a variety of fungal and yeast infections [20] with high-safety profile and has been used as rinsing solution for treatment of oral candidiasis [21, 22]. Hence, we hypothesized that PI acting together with fluconazole could reduce the bacterial and fungal carriage to disrupt mixed *S. mutans-C. albicans* biofilms on teeth, which may lead to a practical antimicrobial therapy for clinical use. Using *in vitro* and *in vivo* biofilm models, we observed that fluconazole and PI alone had only moderate antifungal or antibacterial activity. However, the combination of agents eradicated *C. albicans* carriage and disrupted mixed-biofilm formation without increasing bacterial killing activity *in vivo*. Unexpectedly, the inclusion of PI boosted antifungal efficacy of fluconazole by potently disrupting the assembly of a protective bacterial exopolysaccharides (EPS) matrix through inhibition of α-glucan synthesis by *S. mutans* exoenzyme (GtfB) bound on the fungal surface. Mechanistically, we found that the GtfB-derived EPS produced *in situ* act as "drug trapping matrix" adsorbing the antifungal agent, while inactivation or degradation of α-glucans re-established *Candida* susceptibility to fluconazole. Our findings reveal that EPS produced by the bacterial counterpart can amplify *C. albicans* drug tolerance, indicating that EPS-targeting approaches may be required for optimal antifungal efficacy in the context of cross-kingdom biofilms.

## Materials and methods

### Microorganisms and growth conditions

*Candida albicans* SC5314 (a well-characterized fungal strain) and *Streptococcus mutans* UA159 serotype c (an established cariogenic dental pathogen and well-characterized EPS producer) were used to generate single-species or mixed-species biofilms. *S. mutans gtfB*-defective mutant strain (*gtfB*Δ), and *C. albicans* matrix (mannan–glucan complex)-defective mutant (*kre5*ΔΔ) and SN152 (reference) strains were also used for biofilms assays. For inoculum preparation, *C. albicans* (yeast form) and *S. mutans* cells were grown to mid-exponential phase (optical density at 600 nm (OD_600_) of 0.65 and 0.5, respectively) in ultrafiltered (10-kDa molecular-mass cutoff membrane; Millipore, MA, USA) tryptone-yeast extract broth (UFTYE; 2.5% tryptone and 1.5% yeast extract) with 1% (wt/vol) glucose at 37 °C and 5% CO_2_ as described previously [13, 15].

### *In vitro* biofilm model

**Fig. 1:**
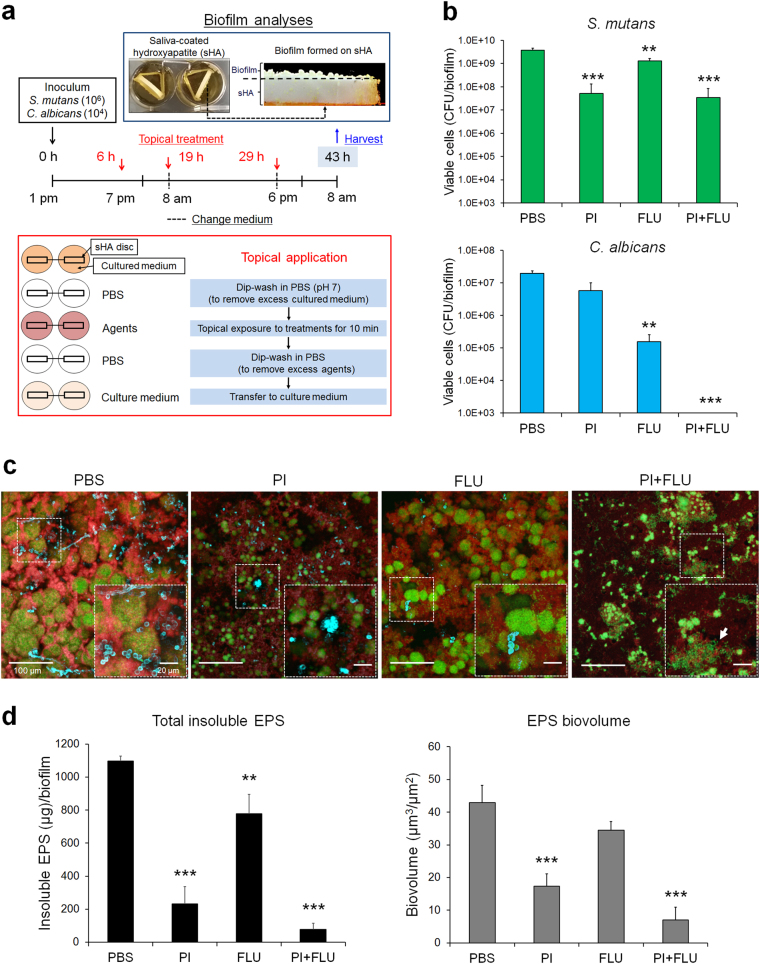
Influence of topical treatments of PI and fluconazole on mixed-kingdom biofilm formation *in vitro*. sHA biofilm model and topical treatment regimen (**a**). Viable cells (CFU) recovered from biofilms following treatments with PBS (vehicle control), povidone iodine (PI at 2% (vol/vol)), fluconazole (FLU at 0.2% (wt/vol)) and PI + FLU (*n* = 8) (**b**). Representative confocal images of mixed species biofilms following treatments; bacterial cells are labeled with SYTO 9 (green), fungal cells with concanavalin A-tetramethylrhodamine (blue) and EPS α-glucan with Alexa Fluor 647 (red) (**c**). White dot-lined box indicates the close-up images of selected area while arrow indicates disorganized microcolonies with sparser EPS accumulation. Total amount of insoluble EPS glucan (as determined via polysaccharides extraction/fractionation and colorimetric quantification; [24]) and total EPS-biovolume (as determined via computational analysis of confocal images of intact biofilms using COMSTAT) (*n* = 8) in each of the treated biofilms (**d**). Data represent mean ± s.d. The quantitative data were subjected to analysis of variance (ANOVA) in the Tukey’s HSD test for a multiple comparison. Values are significantly different from each other at **P < 0.01, ***P < 0.001 (**b**, **d**)

Biofilms were formed using our saliva-coated hydroxyapatite (sHA) disc model as detailed previously [13–15]. Briefly, sHA discs were vertically suspended in a 24-well plate using a custom-made disc holder, and inoculated with approximately 2 × 10^6^ (colony-forming units (CFU)/ml) of *S. mutans* and/or 2 × 10^4^ (CFU/ml) of *C. albicans* (yeast cells) at mid-exponential growth phase in 2.8 ml (per well) UFTYE (pH 7.0) containing 1% (wt/vol) sucrose at 37 °C under 5% CO_2_; this proportion of the microorganisms is similar to that found in saliva samples from children with ECC [23]. The test agents, PI (2% vol/vol) and fluconazole (0.2% wt/vol), were prepared in PBS (pH 7.2), and topically applied to the biofilms three times (at 6, 19 and 29 h) with 10-min exposure (see Fig. 1a). After each treatment, sHA discs were dip-washed in sterile PBS solution to remove excess agents, and then transferred to fresh culture medium. The biofilms formed in each condition were examined using confocal laser scanning microcopy (CLSM) combined with quantitative computational analysis and microbiological assays as described elsewhere [13, 24, 25] (Additional details in Supplementary Materials and Methods).

### GtfB assay

The influence of PI on the activity of surface-adsorbed GtfB was determined as described previously [26]. Briefly, GtfB adsorbed to sHA beads were mixed with PI (at concentrations ranging from 0.0025 to 2%) or PBS control, and then washed to remove excess or unbound material. Then, the treated surface-GtfB was incubated with a [^14^C-glucose]-sucrose substrate (0.2 µCi/ml; 200 mM of sucrose, 40 µM dextran 900, and 0.02% NaN_3_ in buffer consisting of 50 mM KCl, 1 mM CaCl_2_, and 0.1 mM MgCl_2_ at pH 6.5) at 37 °C for 4 h, and the amount of GtfB activity was measured by scintillation counting.

### *In vivo* rodent animal model

**Fig. 2:**
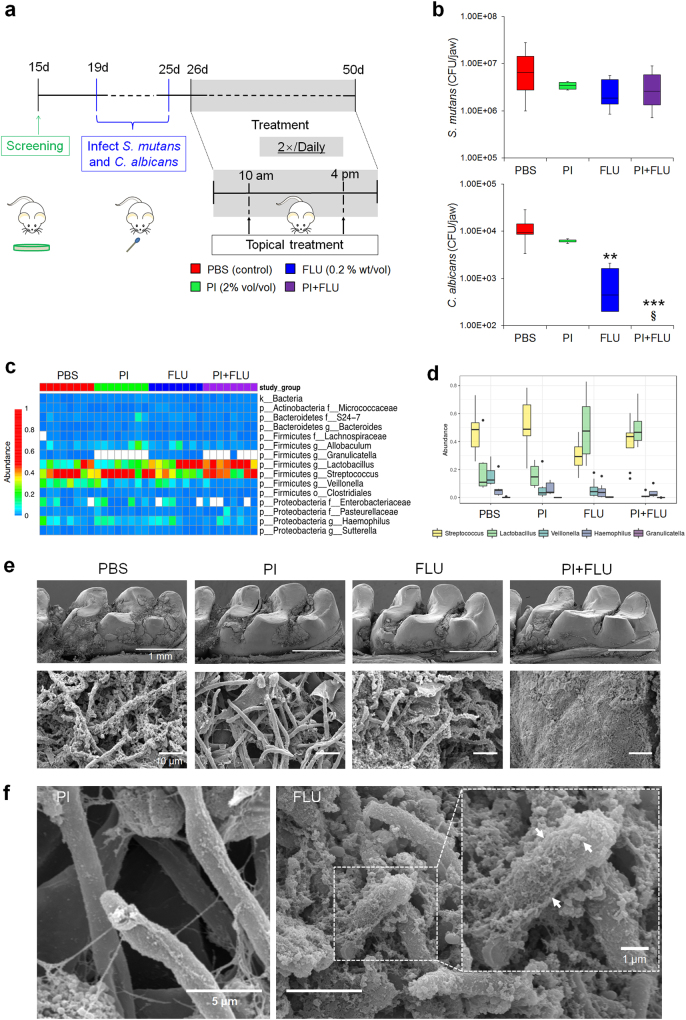
Effect of combination of PI and fluconazole on bacterial–fungal biofilm association *in vivo*. Rodent plaque–biofilm model and topical treatment regimen (**a**). Viable cells (CFU) recovered from plaque biofilms following topical treatments with PBS (vehicle control), povidone iodine (PI at 2% (vol/vol)), fluconazole (FLU at 0.2% (wt/vol)) and PI + FLU (*n* = 8) (**b**). Heatmap of bacterial 16S rRNA gene sequences (**c**) and relative abundance of *Streptococcus*, *Lactobacillus*, *Veillonella*, *Haemophilus* and *Granulicatella* (**d**) (*n* = 8). Representative scanning electron microscopy images of *in vivo* plaque biofilms on smooth tooth surface (**e**) and high-magnification close-up views of selected areas (**f**). White arrows indicate the EPS-like material on the fungal surfaces. In the box whisker plots, whiskers represent minimum and maximum, and the box represents the 25th and 75th percentiles. **P < 0.01 or ***P < 0.001 by two-tailed *t*-test (**b**). §, non-detected

Animal experiments were performed using a well-established rodent model [13, 14]. Briefly, 15 day-old female Sprague–Dawley rat pups were purchased with their dams from Envigo (Madison, WI, USA). The animals were infected by mouth with actively growing (mid-logarithmic) culture of *S. mutans* and *C. albicans* between 19 and 25 days, and their infection (with both organisms) confirmed at 26 days as detailed previously [13]. All the animals were randomly placed into treatment groups, and their teeth were treated topically twice daily with 30 s-exposure using a custom-made applicator (Fig. 2a). The treatment groups were: (1) control (PBS), (2) PI (2% vol/vol), (3) fluconazole (FLU, 0.2% wt/vol), and (4) PI + FLU. Each group was provided the National Institutes of Health cariogenic diet 2000 (TestDiet, St. Louis, MO, USA) and 5% sucrose water ad libitum. At the end of the experimental period, the animals were sacrificed, and the jaws were surgically removed and aseptically dissected. The plaque–biofilm samples were removed and dispersed via sonication and subjected to microbiological analyses as described by Hwang et al. [14]. The structure of mixed-species biofilm was also characterized using a high-resolution environmental scanning electron microscopy (SEM) (Quanta 250 FEG eSEM, FEI, Hillsboro, OR, USA) (Additional details in Supplementary Materials and Methods). This study was reviewed and approved by the University of Pennsylvania Institutional Animal Care and Use Committee (IACUC#805735).

### Metagenomic sequencing

Dispersed plaque sample from jaw was eluted in PBS with cell lysis buffer from a DNeasy kit (Qiagen, Valencia, CA, USA) as described by the manufacturer. After a 60 s vortex, DNA present in the buffer was isolated with the DNeasy kit and quantitated with a spectrophotometer (Tecan, Männedorf, Switzerland). The 27F/338R primer with Golay-barcode in the reverse primer was used to amplify the V1-V2 region of 16S ribosomal DNA (16S rDNA; IDT, Coralville, IA, USA). Details of DNA extraction and PCR amplification are listed in Supplementary Materials and Methods. Sequence data was analyzed with the QIIME pipeline, version 1.9.1 [27]. The forward and reverse reads were joined with no mismatches permitted. Read quality lower than Q29 or more than three consecutive low-quality base calls were discarded. Sequences were clustered into operational taxonomic units at a 97% similarity threshold using the UCLUST method [28]. Taxonomic assignments were obtained based on GreenGenes 16S rRNA gene database v. 13_8 [29]. To test the differences between communities, library vegan and Unifrac distances were used [30, 31]. Bacterial taxon abundances were compared using the Wilcoxon rank sum test. The 16S rRNA gene sequences are available in the NCBI sequence read archive (accession number: SRP133754).

### Impact of *S. mutans* GtfB-derived glucans on antifungal susceptibility

The GtfB was adsorbed to the fungal cells as detailed by Gregoire et al. [17]. Briefly, *C. albicans* yeast cells (~1 × 10^7^/ml) were mixed with saturating amounts of the GtfB (25 μg/ml, 3 U) in adsorption buffer and incubated at 37 °C for 1 h; yeast cell were also incubated in buffer alone (without GtfB) as control. Following adsorption of the enzyme, the cells were centrifuged at 6000×*g*, 4 °C for 10 min and washed twice to remove unbound and loosely bound GtfB. The GtfB-coated yeast cells (or without GtfB; ~1 × 10^4^/ml) were re-suspended in 500 μl of adsorption buffer containing 1% sucrose (wt/vol) and incubated at 37 °C for 4 h. Glucan formation by GtfB adsorbed to the yeast cell surface was visualized by confocal imaging using Alexa Fluor 647-dextran conjugate [17]. For antifungal susceptibility assay, GtfB–glucan coated *C. albicans* cells (or uncoated cells) were mixed with fluconazole (0.2% (wt/vol)) for 18 h. After exposure to fluconazole, supernatants were removed by centrifugation at 14000×*g*, 4 °C for 10 min and the cell pellet washed twice. Re-suspended *C. albicans* cells were plated on the blood agar or Sabouraud dextrose broth agar plate for CFU counting.

### Disruption of EPS-matrix by PI or glucanohydrolases treatment

GtfB-adsorbed *Candida* cells (as described above) was exposed to PI at 0.02% (vol/vol) for 10 min and washed twice to remove excess and then incubated with sucrose substrate for glucan synthesis. The concentration of 0.02% PI can inhibit EPS production on *C. albicans* surface without affecting cell viability as demonstrated in our preliminary experiments. The glucanohydrolases, dextranase (Dex, α-1,6-glucanase; EC 3.2.1.11) and mutanase (Mut, α-1,3-glucanase; EC 3.2.1.59) were used in this experiment to digest α-glucan-matrix produced by GtfB on the fungal surface. Dex produced from *Penicillium sp*. was commercially purchased from Sigma (St. Louis, MO, USA) and Mut produced from *Trichoderma harzianum* were kindly provided by Johnson & Johnson (New Brunswick, NJ, USA). Briefly, the optimized combination of Dex/Mut at 5U and 1U was added to α-glucan coated *C. albicans* and incubated at 37 °C for 1 h. Then, PI-treated or glucanohydrolases-treated cells (and untreated controls) were exposed to fluconazole and the drug susceptibility determined by CFU counting, while the disruption of EPS-matrix (labeled by Alexa Fluor 647-dextran) formed on the fungal surface was evaluated via confocal imaging.

### Live-cell imaging of fluorescent fluconazole uptake in EPS-coated *C. albicans*

*C. albicans* and EPS-coated *C. albicans* were exposed to fluorescent fluconazole labeled with dansyl or Cy5 dyes at 10 or 100 μg/ml (final concentration; [32]), and were placed on glass slides covered with 0.5% agarose bedded glass coverslips. Live-cell images were acquired using an inverted CLSM (SP5-II FLIM, Leica Microsystems, Buffalo Grove, IL, USA) equipped with a 63× (1.4 numerical aperture) oil immersion lens with 5× zoom. The uptake and subcellular co-localization of fluconazole-dansyl/Cy5 and fluorescently labeled EPS-matrix were sequentially scanned using the PicoQuant Sepia II (405 nm) picosecond pulsed diode laser, 488 nm argon laser, 543 nm and 633 nm He-Ne lasers, and the fluorescence emitted was collected with the hybrid detector (424–472 nm for Dansyl-conjugates (fluconazole–Dansyl dye), 475–525 nm for Alexa Fluor 488–dextran conjugates (EPS matrix) and 644–752 nm for Cy5-conjugates (fluconazole-Cy5) or Alexa Fluor 647–dextran conjugates (EPS matrix), respectively. Fluorescence localization was monitored over 60 min of drug exposure using temperature (37 °C) and CO_2_ (5%) controlled chamber. Amira 5.4.1 software (Visage Imaging, San Diego, CA, USA) was used to create renderings of *Z*-stacks (0.2-μm-step size) and visualize localization of fluconazole within EPS-matrix or the cells.

### Sequestration of ^3^H-fluconazole in EPS-coated *C. albicans*

Radiolabeled fluconazole (^3^H, Moravek Biochemicals, Brea, CA, USA) was used to assess drug retention within EPS-matrix surrounding *C. albicans* cells [33]. After formation of α-glucan matrix *in situ* as described above, the EPS-coated cells were incubated with 7 × 10^5^ CPM of ^3^H-fluconazole (final conc. 0.5 μM) in UFTYE medium at 37 °C for 1 h. Unlabeled fluconazole (20 μM) in UFTYE medium was added for additional 15 min incubation. After centrifugation (at 3400×*g*, 4 °C for 20 min), cells were collected and an aliquot of each collected intact cell was saved for scintillation counting as described elsewhere [33] and in the Supplementary Materials and Methods.

### Antifungal susceptibility in single-species *C. albicans* biofilm and mixed-species biofilm co-cultured with *S. mutans gtfB*-defective mutant strain

Biofilms were formed using sHA model as described above. For *C. albicans* single-species biofilm, each disc was inoculated with approximately 2 × 10^4^ (CFU/ml) of *C. albicans* SC5314 or SN152 (reference strain for *kre5∆∆*) or matrix-defective strain (*kre5∆∆*) with or without purified GtfB (15U) in 2.8 ml (per well) UFTYE (pH 7.0) containing 1% (wt/vol) sucrose. For mixed-species biofilms, 2 × 10^4^ (CFU/ml) of *C. albicans* SC5314 and 2 × 10^6^ (CFU/ml) of *S. mutans* UA159 or *gtfB-*defective strain (with or without GtfB supplementation) were co-cultured in sucrose containing UFTYE medium. Fluconazole (0.2% wt/vol) was topically applied to the biofilms three times (at 6, 19 and 29 h; Fig. 1a). The experiments for cell viability, biofilm visualization and ^3^H-fluconazole matrix sequestration were performed as described in the previous sections and in the Supplementary Materials and Methods.

### Statistical analysis

Data represent mean ± standard deviations (s.d). The quantitative data were subjected to analysis of variance (ANOVA) in the Tukey’s HSD test for a multiple comparison. A pairwise comparison was conducted using student’s *t*-test. Statistical analyses were performed using SPSS version 18.0 software (IBM Co., Armonk, NY, USA).

## Results

### PI with fluconazole disrupts fungal–bacterial biofilms *in vitro*

The influence of clinically used concentrations of PI (2%) and fluconazole (0.2%) on mixed-species biofilm development was assessed using a topical treatment regimen (Fig. 1a). sHA was used as tooth-enamel mimetics. PI showed moderate killing effects against *S. mutans* (~2-log reduction of viable cells (CFU), *P* < 0.001), and limited antifungal activity against *C. albicans* (0.5-log reduction vs. PBS control). Conversely, fluconazole exposure resulted in 2-log CFU reduction of *C. albicans* (vs. PBS, *P* < 0.001), while displaying negligible antibacterial activity against *S. mutans*. Notably, topical treatment of PI and fluconazole in combination completely eliminated *C. albicans* viable counts within mixed biofilm, but showed similar killing efficacy against *S. mutans* (vs. PI alone; Fig. 1b). In single-species biofilms, PI alone and PI with fluconazole also displayed comparable antibacterial activity (Supplementary Figure S1a), while fluconazole was devoid of any inhibitory effects against *S. mutans* (Supplementary Figure S1a and b).

To further examine the impact of the treatments on the biofilm structural organization, we performed detailed confocal imaging by fluorescently labeling the bacterial, fungal and α-glucan EPS components (Fig. 1c). Representative images showed that control (PBS-treated) biofilms harbored yeast and hyphal forms of *C. albicans* (in blue) associated with *S. mutans* clusters (or microcolonies; in green) and an abundant EPS matrix (in red). PI-treated biofilms contained smaller bacterial microcolonies and mostly yeast cells of *Candida*. In contrast, fluconazole treatment visibly reduced fungal presence (some yeast cells but no hyphae) without causing major effects on *S. mutans* microcolony. However, biofilms treated with combination of PI and fluconazole was devoid of *C. albicans* cells and contained smaller microcolonies with disorganized structure (see arrow). Interestingly, we also noted that PI or PI + fluconazole treatment showed sparser accumulation of EPS (Fig. 1c). Biochemical quantification of water-insoluble EPS (colorimetric assay) and quantitative computational analysis (COMSTAT) revealed significant reduction in the amount and biovolume of EPS in PI, and particularly in PI + fluconazole biofilm treatment (vs. PBS, *P* < 0.001, Fig. 1d), consistent with confocal imaging data. Together, the data indicate that combination of PI and fluconazole can substantially enhance antifungal activity of fluconazole, while also disrupting the EPS-matrix formation and *S. mutans* microcolony structure, thereby reducing the bulk and density of infection.

### Combination of PI and fluconazole eradicate *C. albicans* carriage in plaque–biofilms *in vivo*

Next, we assessed the anti-biofilm efficacy of topical application of PI and fluconazole *in vivo* using a well-established rodent plaque biofilm model [13, 14] (Fig. 2a). Following establishment of co-infection, the animals were treated twice daily with the agents for 3 weeks using brief topical exposure (30 s) to simulate clinical use of antimicrobials. Microbiological analysis showed that combination of PI and fluconazole completely prevented *C. albicans* carriage within plaque biofilm on the tooth surface. In contrast, topical treatment of PI alone was unable to reduce the number of viable *C. albicans* cells while fluconazole exhibited moderate antifungal effects (~1-log reduction vs. PBS, *P* < 0.01, Fig. 2b). Despite some bacterial reduction, PI-based treatments did not affect significantly the *S. mutans* viability in oral biofilms *in vivo*. The discrepancy of the antibacterial activity of PI observed *in vitro* and *in vivo* may be related to differences between the biofilm models. The rodent model includes exposure to diet, host microbiota and saliva as well as the mechanical–hydrodynamic forces that are typically found in the mouth environment, which can dampen the antibacterial efficacy. Nevertheless, it is important to note that PI was only moderately effective against biofilms *in vitro*, which could explain the limited efficacy *in vivo*.

Since depletion of *C. albicans* by the combination therapy could affect the microbiota composition in plaque–biofilms, we also assessed the impact of treatment on bacteriome profile via metagenomics sequencing. Heatmap of bacterial 16S rRNA gene sequences revealed significant changes of bacterial abundance and proportions following treatment (Fig. 2c). Reduction (fluconazole) or depletion (PI + fluconazole) of *C. albicans* caused significant alterations in the relative abundance (*P* < 0.05) and the proportion of major rodent oral bacteria including *Streptococcus, Lactobacillus, Veillonella, Granulicatella* and *Haemophilus* (Fig. 2d). In particular, we found a negative correlation in the relative abundance between *Lactobacillus* and *Streptococcus* following depletion of *C. albicans* (*r*^2^ = 0.68, Supplementary Figure S2), consistent with previous observations that *Lactobacillus* and *C. albicans* are antagonistic [10, 34], while *Streptococcus* has symbiotic interactions with *C. albicans* [3, 13].

The effect of topical application of the agents on plaque biofilm formation was also examined by SEM (Fig. 2e,f). In the PBS-treated group, we observed abundant plaque biofilms over the tooth surface and many *C. albicans* cells were surrounded by EPS-like matrix. In contrast, *C. albicans* cells were undetected in plaque biofilms treated with the combination of PI and fluconazole (Fig. 2e). Interestingly, PI treatments visibly reduced the amounts of EPS-like materials on the fungal surface, while biofilms-treated with fluconazole alone harbored *C. albicans* covered by extracellular matrices (see arrows; Fig. 2f).

Collectively, the *in vitro* and *in vivo* biofilm data showed that combination of PI and fluconazole completely eradicated *C. albicans* without enhancing killing activity against *S. mutans*. Interestingly, PI-based treatments also reduced the amounts of EPS in mixed-biofilm *in vitro*, while *C. albicans* cells with less extracellular substances surrounded them were found *in vivo*. Thus, we investigated how PI could enhance the antifungal efficacy and whether bacterially-derived EPS on the fungal surface can modulate antifungal tolerance.

### PI enhanced the antifungal susceptibility via inhibition of EPS-matrix formation

**Fig. 3:**
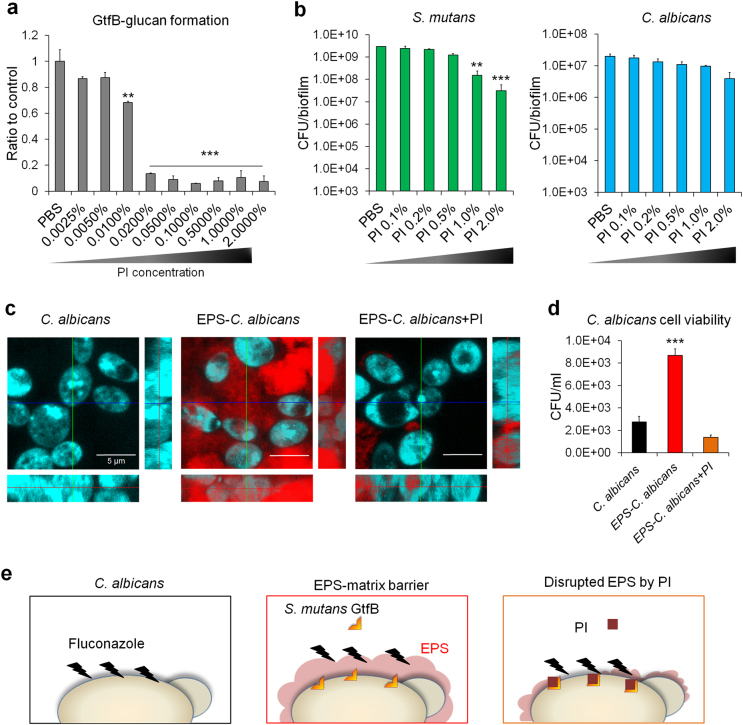
Inhibition of *S. mutans* GtfB-derived EPS (α-glucan) synthesis by PI enhances susceptibility of *C. albicans* to fluconazole. Inhibitory activity of PI on surface-absorbed GtfB. Data represent relative ratio to control (PBS, defined as 1) (**a**). Microbial cell viability (*S. mutans* or *C. albicans*) within biofilm (**b**). Confocal images of uncoated, EPS-embedded and EPS-disrupted *C. albicans* (fungal cells are depicted in blue; EPS are shown in red) (**c**) and cell viability of *C. albicans* following treatment with fluconazole (**d**). Proposed “EPS-shielding model” for antifungal resistance afforded by the presence of *S. mutans* GtfB-derived α-glucans matrix as well as re-establishment of antifungal susceptibility through disruption of GtfB–glucan (**e**). Data represent mean ± s.d. (*n* = 6). The quantitative data were subjected to analysis of variance (ANOVA) in the Tukey’s HSD test for a multiple comparison. Values are significantly different from each other at **P < 0.01 or ***P < 0.001 (**a**, **b**, **d**)

Since this fungal–bacterial partnership relies on α-glucans formed by GtfB bound onto *C. albicans* and iodine affects Gtfs enzymatic activity (Supplementary Figure 3) [35], we hypothesized that PI can reduce the EPS synthesis *in situ* and thereby expose the fungal cells against fluconazole. Results from dose–response experiments showed that PI potently inhibited the activity of surface-adsorbed GtfB and glucan synthesis (>80% inhibition) even at concentrations as low as 0.02% (vol/vol) (*P* < 0.001, Fig. 3a). Interestingly, 50–100 times higher concentrations of PI (1 and 2%) were required to reduce *S. mutans* viability within mixed-biofilm (Fig. 3b); as expected PI was devoid of antifungal activity. Hence, PI appears to be a more potent GtfB inhibitor than an antibacterial agent, and thus could modulate glucan production that is critical for *S. mutans*–*C. albicans* co-existence within biofilms.

We next investigated whether PI acting as a GtfB inhibitor can disrupt EPS production on *Candida* cell surface, and thereby enhance the antifungal susceptibility. Confocal imaging confirmed α-glucan EPS (in red) surrounding *C. albicans* cells (in blue), whereas PI treatment drastically diminished the EPS synthesis on the cell surface (Fig. 3c). Then, the uncoated, EPS-coated and PI-disrupted EPS *C. albicans* cells were treated with fluconazole and the number of viable fungal cells were determined (Fig. 3d). The results showed that EPS-enmeshed *C. albicans* was significantly less susceptible to fluconazole than uncoated *C. albicans* (*P* < 0.001). However, EPS-disrupted *C. albicans* exhibited similar antifungal susceptibility to that of uncoated *Candida*, suggesting that the bacterial-derived EPS may act as a protective barrier for the fungal cells (Fig. 3e).

### *S. mutans*-derived EPS matrix prevents fluconazole uptake to *C. albicans*

**Fig. 4:**
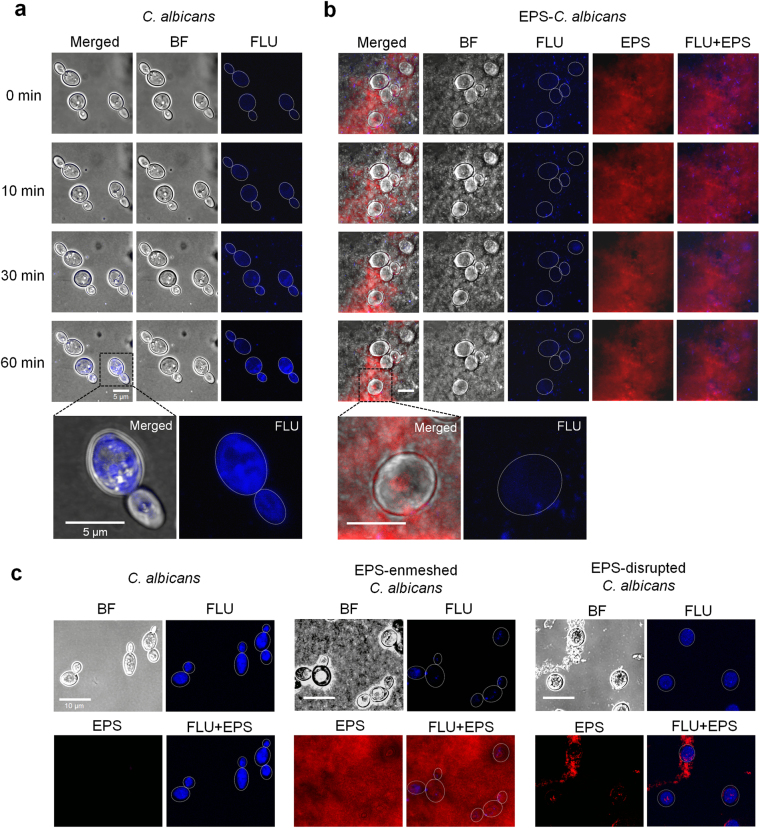
Influence of bacterial α-glucan matrix and PI treatment on fluconazole uptake by *C. albicans* cells. Uptake of fluconazole in *C. albicans* without (**a**) and with (**b**) α-glucan EPS-matrix was determined by time-lapse live cell imaging using dansyl-conjugated fluconazole (depicted in blue). Fluconazole uptake by *C. albicans*, EPS-embedded *C. albicans* and EPS-disrupted *C. albicans* via PI treatment (**c**). Uptake of pulsed fluconazole was monitored using confocal microscopy within temperature and CO_2_ controlled chamber for 60 min. At least three independent biological replicates were performed. BF bright-field microscopy

In order to determine the role of GtfB-derived EPS in modulating antifungal drug susceptibility, we investigated whether *S. mutans*-derived EPS matrix can prevent fluconazole uptake by *C. albicans*. A new generation of fluorescent azole probes, including fluconazole, were recently developed allowing precise visualization of cellular uptake and subcellular localization of the antifungals [32]. Real-time live-cell fluorescence imaging was used to assess whether *S. mutans* EPS-matrix can prevent the uptake of dansyl-labeled or Cy5-labeled fluconazole by *C. albicans*. Time-lapse imaging reveals that the uptake and accumulation of fluconazole (in blue) inside of *C. albicans* cells occurred within 10–30 min (Fig. 4a). In contrast, fluconazole uptake and subcellular localization was substantially reduced in EPS-enmeshed *C. albicans*, showing either no or sparse fluorescent signal after 60 min (Fig. 4b). Detailed three-dimensional analysis of individual fungal cell using projection and orthogonal views revealed that fluconazole (in blue) was fully transported and localized inside the uncoated *C. albicans*, whereas no fluorescent signal was detected neither on the cell wall nor intracellularly across the single-cell structure (Supplementary Figure S4a and b). Conversely, merged confocal images shows fluconazole localized extracellularly and associated with the EPS matrix, indicating that the surrounding α-glucan might be impeding intracellular access. Thus, it is conceivable that the uptake of fluconazole can be facilitated by PI treatment through inhibition of EPS-matrix formation. The confocal images clearly show that reduction of EPS-matrix formation results in enhanced fluconazole accumulation inside the cell, in a similar manner to that observed in uncoated *C. albicans* (Fig. 4c). Therefore, PI can enhance *Candida* susceptibility to fluconazole by disrupting the protective EPS matrix barrier through potent inhibition of GtfB–glucan synthesis.

### Effect of glucanohydrolases on antifungal susceptibility of glucan-enmeshed *C. albicans*

**Fig. 5:**
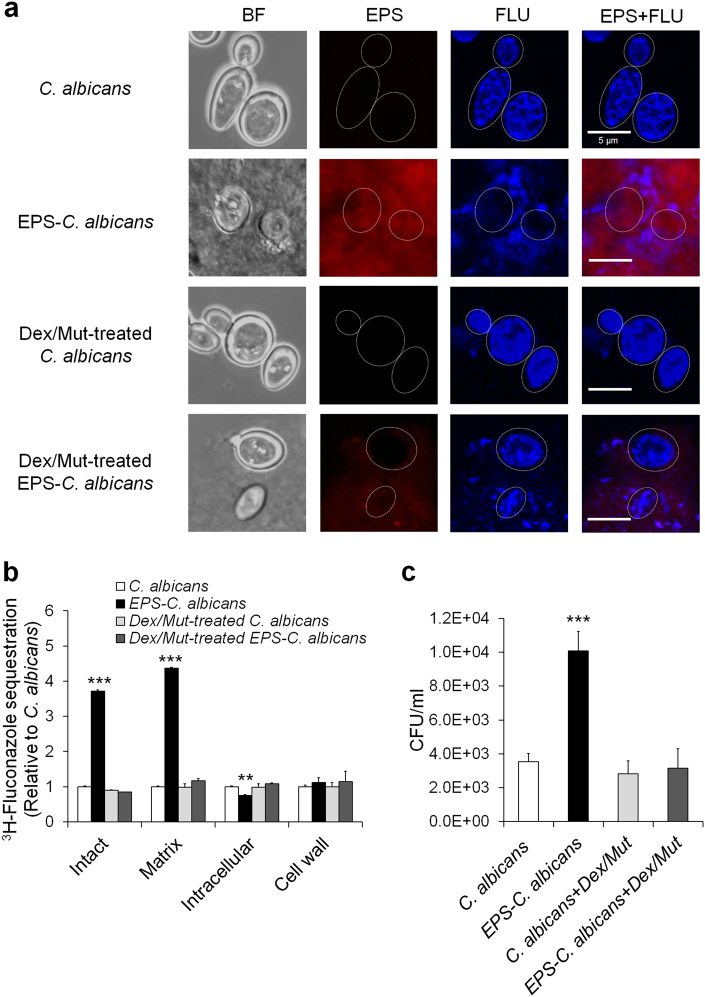
Fluconazole sequestration and antifungal susceptibility in α-glucan-embedded *C. albicans* cells with and without EPS degradation. Dextranase (Dex, 5 U) and mutanase (Mut, 1 U) were used to enzymatically digest the α-glucan EPS-matrix formed on the fungal surface. High resolution confocal images show fluconazole localized within the mitochondria or bound within α-glucan (**a**). Fluconazole sequestration in EPS-embedded and EPS-degraded *C. albicans* (**b**). The values of ^3^H-fluconazole were compared with those of uncoated *C. albicans* and expressed as relative ratio. Cell viability of EPS-embedded and EPS-degraded *C. albicans* following fluconazole treatment (**c**). Data represent mean ± s.d. (*n* = 6). The quantitative data were subjected to analysis of variance (ANOVA) in the Tukey’s HSD test for a multiple comparison. Values are significantly different from each other at **P < 0.01 or ***P < 0.001 (**b**, **c**)

Results from live-cell imaging suggest that α-glucans formed on *C. albicans* may confer protection against fluconazole. To further understand the mechanisms by which GtfB-derived EPS impact fluconazole uptake by *C. albicans*, we investigated whether the EPS-matrix can bind and sequester fluconazole using both fluorescently labeled and radiolabeled-fluconazole. We initially assessed the distribution pattern of fluorescent fluconazole using high-resolution confocal imaging (Fig. 5a). After 60-min exposure, fluorescence was evident within the cells and exhibited a distinctive subcellular distribution pattern in uncoated *C. albicans*, consistent with co-localization of the probe with the mitochondria (Supplementary Figure 5) [32]. In contrast, fluconazole clearly localized outside of cells when EPS was present, forming defined fluorescent patches that were associated with the α-glucans surrounding the fungal cell, indicating that fluconazole retained within the matrix. To further verify this, we used radiolabeled ^3^H-fluconazole to determine whether α-glucans matrix could sequester the drug [33]. Following fluconazole exposure, a sequential fractionation of matrix, cell wall and cellular components of EPS-enmeshed *C. albicans* was performed, and the radiolabeled drug measured via scintillation counting. Consistent with fluorescence imaging, ^3^H-fluconazole sequestration assay indicates that EPS-matrix retained fluconazole (4.5-fold increase vs. uncoated *C. albicans*, *P* < 0.001) (Fig. 5b).

Since dextranase (Dex) and mutanase (Mut) can specifically digest GtfB-derived α-glucans by targeting α-(1 → 6) and α-(1 → 3) glycosyl linkages [36], we examined whether glucan degradation in EPS-enmeshed *C. albicans* can facilitate fluconazole uptake and re-establish antifungal susceptibility. Fluorescence images showed that the matrix was effectively digested with only residual α-glucans sparsely detected. Conversely, EPS digestion restored fluconazole uptake and intracellular accumulation within the *Candida* cells, while some of the drug outside of the cell was associated with the remaining glucans (Fig. 5a). These findings were corroborated with ^3^H-fluconazole sequestration data (Fig. 5b). Furthermore, we also determined the viability of *C. albicans* following the same treatments. Based on CFU recovery, EPS-enmeshed *C. albicans* exhibited enhanced tolerance to fluconazole compared to uncoated *C. albicans* (Fig. 5c). As expected, EPS-digested *C. albicans* showed similar antifungal susceptibility to that of uncoated *Candida*, while Dex/Mut treatment was without effects on fungal viability.

### *S. mutans*-derived GtfB modulates antifungal protection within biofilms

**Fig. 6:**
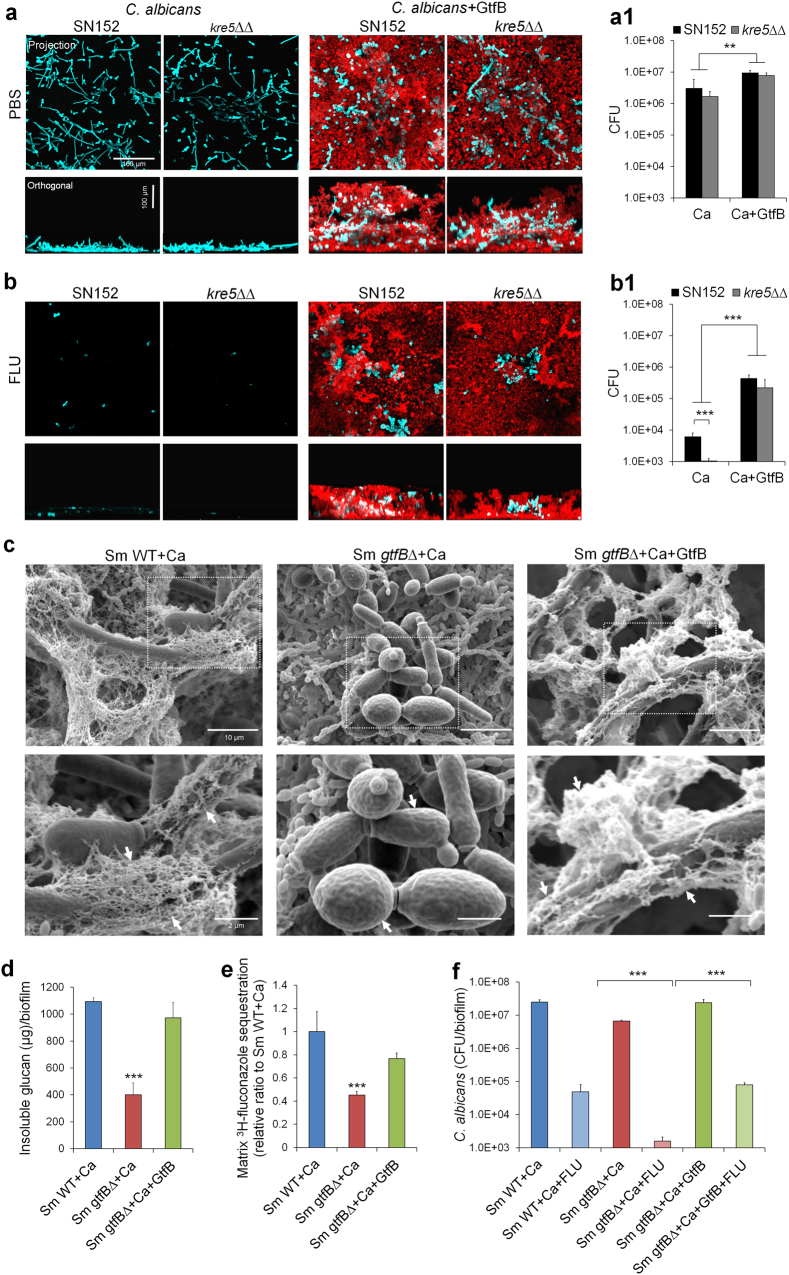
GtfB α-glucan modulates antifungal susceptibility during *C. albicans* single or mixed-species biofilm formation. *C. albicans* single-species (wild type vs. *kre5∆∆*) or mixed-species biofilms co-cultured with *S. mutans gtfB∆* were formed in UFTYE (pH 7.0) containing 1% (wt/vol) sucrose with or without the addition of purified GtfB (15 U). PBS or fluconazole (0.2% wt/vol) were topically applied to the biofilms as described in Fig. 1. Representative confocal images of single-species biofilms treated with PBS (**a**) or fluconazole (FLU) (**b**). Fungal cells are labeled with concanavalin A-tetramethylrhodamine (blue) while EPS α-glucans were labeled with Alexa Fluor 647 (red). Cell viability (CFU) following treatment with PBS (**a1**) or FLU (**b1**). Representative SEM images of mixed-species biofilms (**c**). *S. mutans* wild type with *C. albicans* (Sm WT + Ca), *S. mutans gtfB*-defective mutant with *C. albicans* (Sm *gtfB*Δ + Ca) and *S. mutans gtfB*Δ with *C. albicans* supplemented with purified GtfB (Sm *gtfB*Δ + Ca + GtfB). Arrows indicate either presence (far-right and far-left panels) or absence (middle-panel) of EPS-matrix on the fungal cell surfaces. Total insoluble EPS glucan as determined colorimetrically as described in Supplementary Materials and Methods (**d**). Fluconazole matrix sequestration assays in mixed-species biofilms (**e**); the values of ^3^H-fluconazole were compared with those of Sm WT + Ca and expressed as relative ratio. Cell viability of *C. albicans* in mixed-species biofilm co-cultured with *S. mutans* wild-type (WT) or *S. mutans gtfB*-defective strain with or without GtfB either treated with fluconazole or PBS (**f**). Data represent mean ± s.d (*n* = 6). The quantitative data were subjected to analysis of variance (ANOVA) in the Tukey’s HSD test for a multiple comparison. Values are significantly different from each other at **P < 0.01 or ***P < 0.001 (**a1**,** b1**, **d**, **e**). ***P < 0.001 by two-tailed *t*-test (**f**)

We next sought to investigate the role of GtfB in mediating C. *albicans* susceptibility to fluconazole within single and mixed-species biofilms using biochemical and genetic approaches. Initially, we examined whether the addition of purified GtfB enzyme affects the antifungal resistance of single-species *C. albicans* biofilms. Fungal biofilms were grown on sHA discs in sucrose-containing medium supplemented with GtfB (15 U) or without the enzyme, and topically treated with fluconazole as described previously. Confocal images and viable cell counting revealed that the presence of GtfB enhanced *C. albicans* (in blue) colonization and carriage onto sHA surface, forming biofilms with an abundant α-glucan EPS matrix (in red) (vs. *C. albicans* without GtfB supplementation, *P* < 0.01) (Fig. 6a, a1). Concomitantly, we also observed that the presence of GtfB significantly enhanced *C. albicans* biofilm resistance to fluconazole, while the fungal cells without the bacterial exoenzymes were susceptible to antifungal treatment essentially preventing biofilm development (*P* < 0.001, Fig. 6b, b1). Since fungal-derived matrix polysaccharides (e.g. mannan and β-glucan) are also linked with drug resistance in *C. albicans* [33, 37], we investigated whether GtfB α-glucan can provide protection to a matrix-defective strain (*C. albicans kre5∆∆*). *C. albicans kre5∆∆* was more susceptible to fluconazole treatment compared to wild type (Fig. 6b1), consistent with previous findings despite differences in the biofilm model and culturing conditions [33]. Strikingly, however, the matrix-defective fungal strain (*C. albicans kre5∆∆*) and *C. albicans* wild type (SN152) were both equally resistant to antifungal killing when supplemented with GtfB (*P* < 0.001, Fig. 6b1). We found that GtfB can bind to *C. albicans kre5∆∆* in active form (Supplementary Figure 7) resulting in similar number of fungal cells and overall structure (vs. wild type) with large amounts of α-glucan formed *in situ* (Fig. 6a, b). Furthermore, this protective mechanism provided by the bacterial EPS was recapitulated in mixed-biofilms treated with fluconazole whereby *C. albicans kre5∆∆* and *C. albicans* SN152 exhibited comparable counts of viable fungal cells (CFU) when co-cultured with *S. mutans* (Supplementary Figure S6a) (*P* > 0.05).

**Fig. 7:**
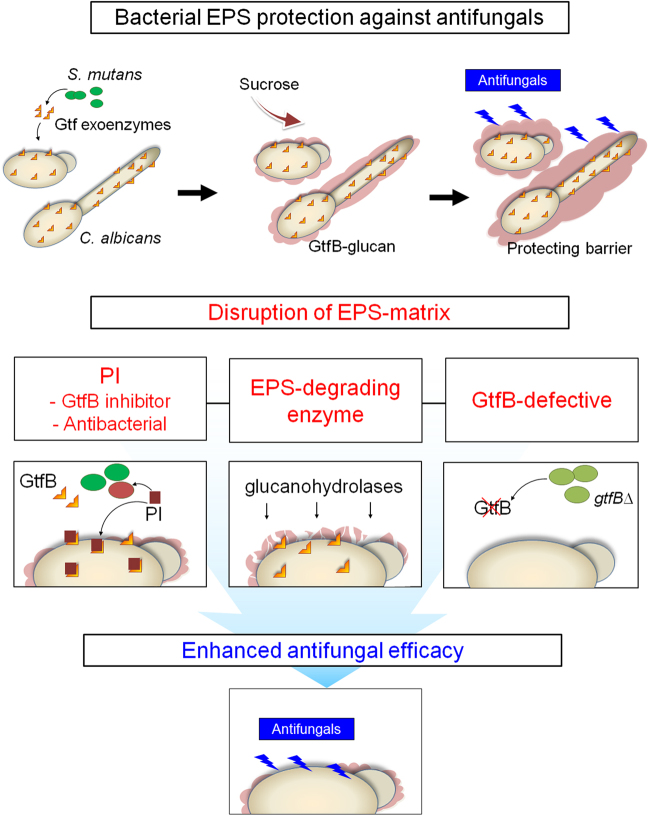
Proposed schematic model illustrating bacterial EPS-mediated antifungal tolerance and disruption of the protective matrix for enhanced antifungal efficacy

Next, we reasoned that *S. mutans* lacking *gtfB* (*gtfB*∆) would lead to defective “drug shielding matrix” for *C. albicans* within mixed biofilms, enhancing antifungal susceptibility to fluconazole. The data showed that *C. albicans* co-cultured with *gtfB*∆ mutant resulted in altered biofilm architecture with sparse matrix covering the *C. albicans* cell-surface, as evidenced in the SEM close-up images (see arrows; Fig. 6c) and significantly less insoluble α-glucan amounts (*P* < 0.001, Fig. 6d). These observations were further expanded by ^3^H-fluconazole sequestration assay, whereby mixed wild-type biofilms contained higher levels of matrix-bound fluconazole than those formed with GtfB-defective strain (*P* < 0.001, Fig. 6e); as expected, GtfB supplementation restored fluconazole sequestration in the matrix of mixed biofilms with *gtfB*∆ mutant. The alterations in the matrix assembly by *gtfB*∆ mutant enhanced killing efficacy of fluconazole against *C. albicans* when compared to mixed-biofilms formed with the parental *S. mutans* strain (*P* < 0.001, Fig. 6f). Conversely, supplementation with purified GtfB restored the mixed biofilm EPS-matrix phenotype and architecture in the presence of the *gtfB*∆ mutant (Fig. 6c, d), while concurrently increasing the antifungal resistance of *C. albicans* (Fig. 6f). Therefore, targeting this protective mechanism afforded by the bacterial EPS can enhance the efficacy of antifungal treatment and prevent *Candida* carriage to disrupt this cross-kingdom biofilm interaction (Fig. 7).

## Discussion

Current chemical modalities to treat biofilm-associated infections are primarily aimed at targeting individual bacterial or fungal components despite increasing evidence showing that polymicrobial interactions can mediate biofilm virulence and drug resistance. Laboratorial data have shown that monotherapies have limited efficacy to treat cross-kingdom biofilms [4, 5]. Here, we show that the combination of topical antifungal fluconazole with PI can completely suppress *C. albicans* carriage and disrupt mixed-kingdom biofilm development *in vivo*. Contrary to our expectations, the enhanced antifungal efficacy was achieved without increasing bacterial killing. Rather, PI boosted fluconazole efficacy by potently disrupting the assembly of bacterial-derived EPS matrix through inhibition of EPS-producing exoenzymes. Further analyses revealed a protective mechanism whereby exoenzymes from *S. mutans* (GtfB) bound to *Candida* surface produced α-glucans *in situ*, “wrapping” the fungal cells with a dense layer of polymeric matrix (Supplementary Figure 8). We found that the GtfB-derived EPS directly bound and sequestered fluconazole, impeding cellular drug uptake and thereby enhancing antifungal drug tolerance. Conversely, inhibition of GtfB activity by PI, enzymatic degradation of α-glucans or co-culturing with *gtfB*-inactivated *S. mutans* restored the antifungal susceptibility of *C. albicans*. Thus, we provide compelling evidence that targeting bacterial EPS is a key factor for enhanced antifungal drug efficacy in the context of mixed kingdom biofilms (Fig. 7).

The presence of self-produced EPS-matrix (mannans and β-glucan) has been recognized as an important virulence attribute associated with antimicrobial drug resistance in fungal biofilms [37–39]. Highly branched β-1,3-glucan matrix in *C. albicans* single-species biofilms has been shown to prevent drugs from reaching their own cellular target through drug sequestration *in vitro* [33]. However, previous observations suggest that in mixed cultures the bacterial counterpart could contribute to the enhanced antifungal drug resistance through unknown factors [7, 40, 41]. Our data reveal that EPS-producing bacterial GtfB exoenzymes can directly modulate antifungal drug tolerance both at a single-cell level and within multicellular biofilms, even if *C. albicans* is defective in producing its own protective matrices. Prior investigations have shown that α-glucans formed on the surrogate fungal surface markedly enhances co-adhesion between *S. mutans* and *C. albicans*, while boosting fungal colonization of tooth surfaces [13, 14, 42]. Once together, *S. mutans* provides benefits to *C. albicans* by cross-feeding sucrose break-down products (glucose) for fungal utilization and growth [43, 44] while influencing yeast-hyphae transition via chemical interactions [45–47]. Importantly, the presence of *C. albicans* activates GtfB production by *S. mutans* creating an EPS-producing loop [13, 15]. We found that this partnership provides advantages for fungi survival and drug tolerance in mixed-biofilms as the bacterial EPS forms a “drug trapping matrix” that prevents uptake and subcellular localization of antifungal agents. Our data establish an intriguing physicochemical mechanism whereby a bacterially-produced exoenzyme functions on the surface of another kingdom to directly mediate drug tolerance.

The findings that *Candida* depletion in the animal model can alter the bacterial composition of biofilm also raise new questions related to therapeutic implications of *Candida*-bacterial interactions *in vivo.* *C. albicans* has been shown to enhance antibacterial drug resistance by similarly providing a protective fungal β-1,3-glucan barrier that increases tolerance of staphylococci to vancomycin [1, 4, 48]. Interestingly, we observed that neither presence nor depletion of *C. albicans* affected the bacterial killing of *S. mutans* by PI (Supplementary Figure S1a), while the agent had similar antibacterial activity against *S. mutans* co-cultured with matrix-defective *C. albicans* (Supplementary Figure S6b), suggesting a fungal-matrix independent mechanism in our system. This is consistent with our previous observations that mixed *C. albicans-S. mutans* biofilm contains only small accumulation of fungal β-glucan interspersed within copious amounts of bacterially derived α-glucans in the matrix [13]. Hence, in a mixed-kingdom relationship, the EPS produced by the bacterial counterpart can also protect the fungal organism.

Although PI is a potent GtfB inhibitor and acts concurrently with fluconazole to eliminate *C. albicans*, the agent displays only moderate antibacterial activity against *S. mutans*. Given that *C. albicans* presence can promote *S. mutans* growth within biofilms [15, 43, 44], it will be interesting to assess the long-term effects on bacterial survival or whether inclusion of more potent antibacterial agents can also eradicate *S. mutans* within polymicrobial biofilms following *Candida* depletion *in vivo*. A further question worth pursuing using the animal model is how selective fungal removal or re-infection can affect the bacteriome metabolism, inter-species interactions and biofilm virulence over time, particularly on the severity of dental caries. Concomitantly, additional bacterial-derived and fungal-derived chemical factors or matrix components can be identified dynamically, and the role of transient chemical and physical interactions can be interrogated. Additional studies to address these questions may lead to better understanding of cross-kingdom pathogenesis and drug resistance mechanisms.

Our observations also suggest future strategies to overcome the limitation of current antifungal monotherapies against cross-kingdom biofilms. Selective depletion of the fungal pathogen can be achieved by combining existing antifungals with bacterial EPS matrix-targeting agents, which may help develop feasible clinical applications. Specifically, the simple inclusion of PI can tarnish *C. albicans* carriage on teeth while making them more susceptible to fluconazole. In addition, the identification of GtfB as a key modulator in the antifungal drug susceptibility may lead to proof-of-concept clinical studies to assess the efficacy of readily available GtfB inhibitors (PI) or glucanohydrolases (dextranase/mutanase) in combination with antimicrobials. Altogether, the present study provides new insights to combat antifungal drug tolerance and optimize antibiofilm efficacy in the context of a mixed-kingdom oral biofilm, which may be applicable to other intractable polymicrobial infections.

## Electronic supplementary material

Supplementary Figures

Supplementary Materials and Methods
