# Supplementary material for: Bacterial-derived exopolysaccharides enhance antifungal drug tolerance in a cross-kingdom oral biofilm
Source: ISME J. 2018 Apr 18;12(6):1427–42. doi: 10.1038/s41396-018-0113-1 (PMC5955968; doi:10.1038/s41396-018-0113-1)
Supplement: Supplementary file 1 — Supplementary Figures [file 41396_2018_113_MOESM1_ESM.docx]

**Supplementary Figures**

**Bacterial-derived exopolysaccharides enhance antifungal drug tolerance in a cross-kingdom oral biofilm**

Dongyeop Kim^a^, Yuan Liu^a^, Raphael I. Benhamou^b^, Hiram Sanchez^c^, Áurea Simón-Soro^a^, Yong Li^a^, Geelsu Hwang^a^, Micha Fridman^b^, David R. Andes^c^, Hyun Koo^a^

^a^ Biofilm Research Laboratory, Department of Orthodontics and Divisions of Pediatric Dentistry & Community Oral Health, School of Dental Medicine, University of Pennsylvania, Philadelphia, PA, USA

^b^ School of Chemistry, Raymond & Beverly Sackler Faculty of Exact Sciences, Tel Aviv University, Tel Aviv, Israel

^c^ Departments of Medicine and Medical Microbiology and Immunology, University of Wisconsin, Madison, WI, USA

**
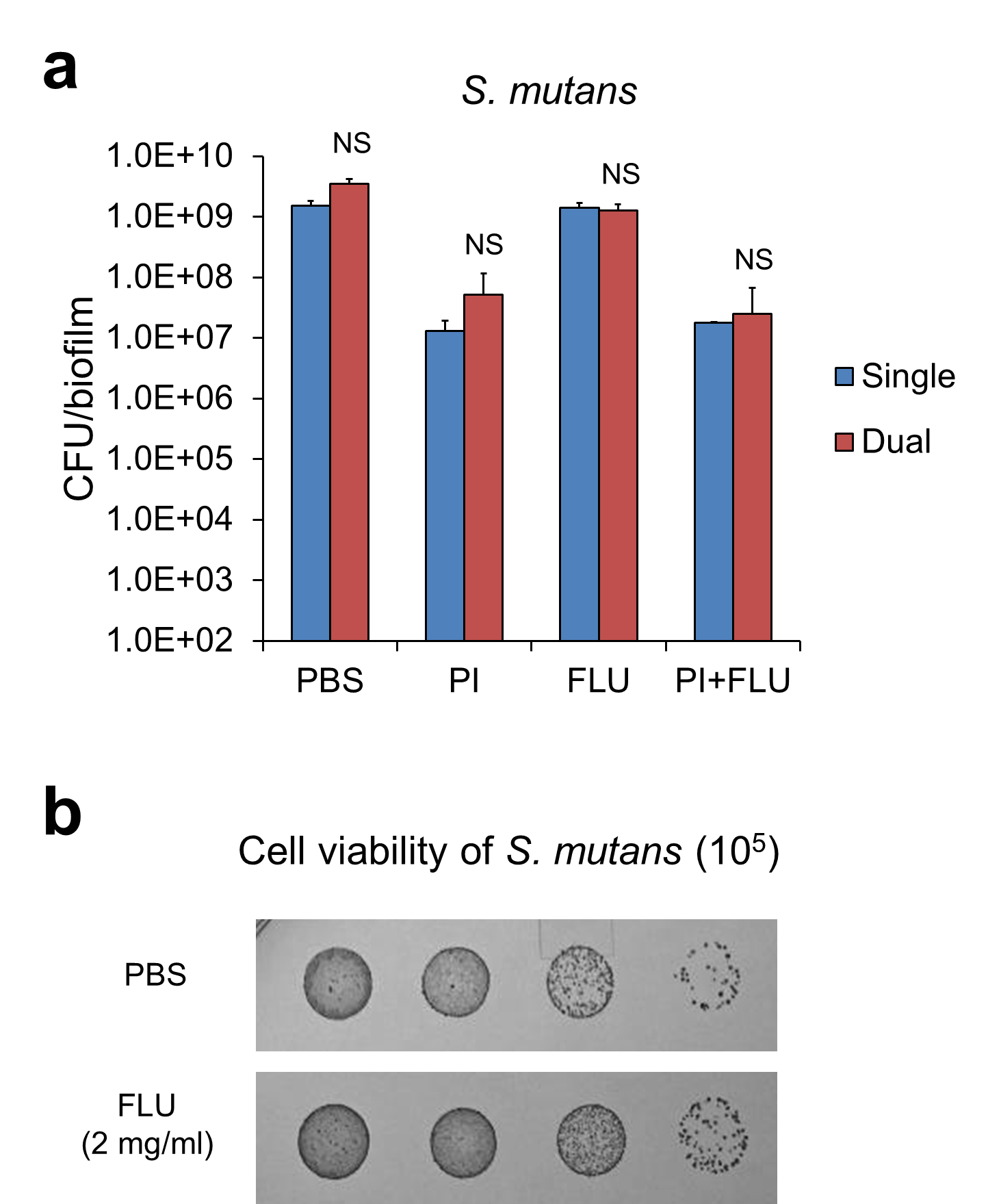
**

**Supplementary Figure 1. Influence of topical treatment of povidone iodine (PI) and fluconazole (FLU) on *S. mutans* single and *S. mutans-C. albicans* mixed-species biofilms *in vitro*.** CFU recovery from biofilms following treatments with PBS (vehicle control), PI (at 2% (v/v)), FLU (0.2% (w/v)) and PI+FLU (n = 4); non-significant differences between single- and dual-species biofilms (*P*>0.05) (**a**). Cell viability of *S. mutans* on fluconazole containing MSA agar plate (**b**). PI displayed similar antibacterial activity in single- and mixed-species biofilms. Fluconazole did not show any inhibitory effects on *S. mutans* biofilm formation (**a**) and cell growth (**b**). Data represent mean ± s.d (n = 4). The quantitative data were subjected to *t*-test. NS, non-significant.

**
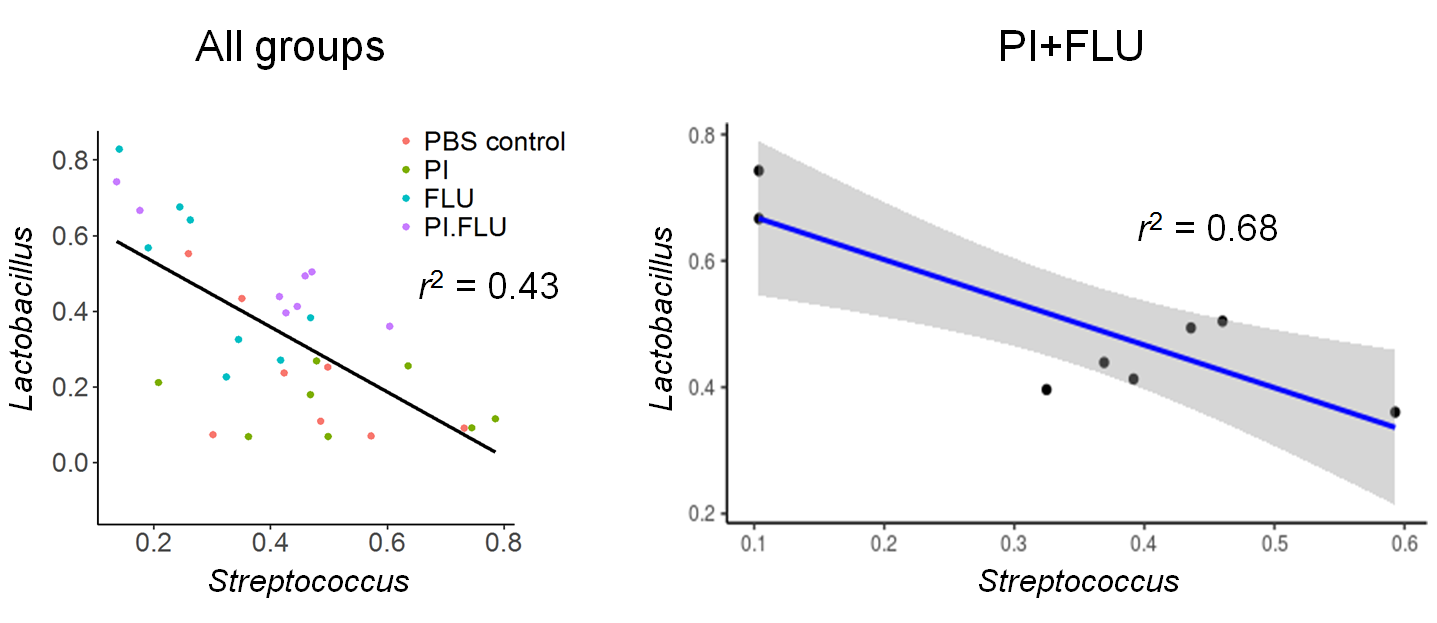
**

**Supplementary Figure 2. Correlation in the relative abundance between *Lactobacillus* and *Streptococcus*.** The data reveal a negative correlation in the relative abundance between *Lactobacillus* and *Streptococcus* following depletion of *C. albicans* by PI+FLU treatment.

**
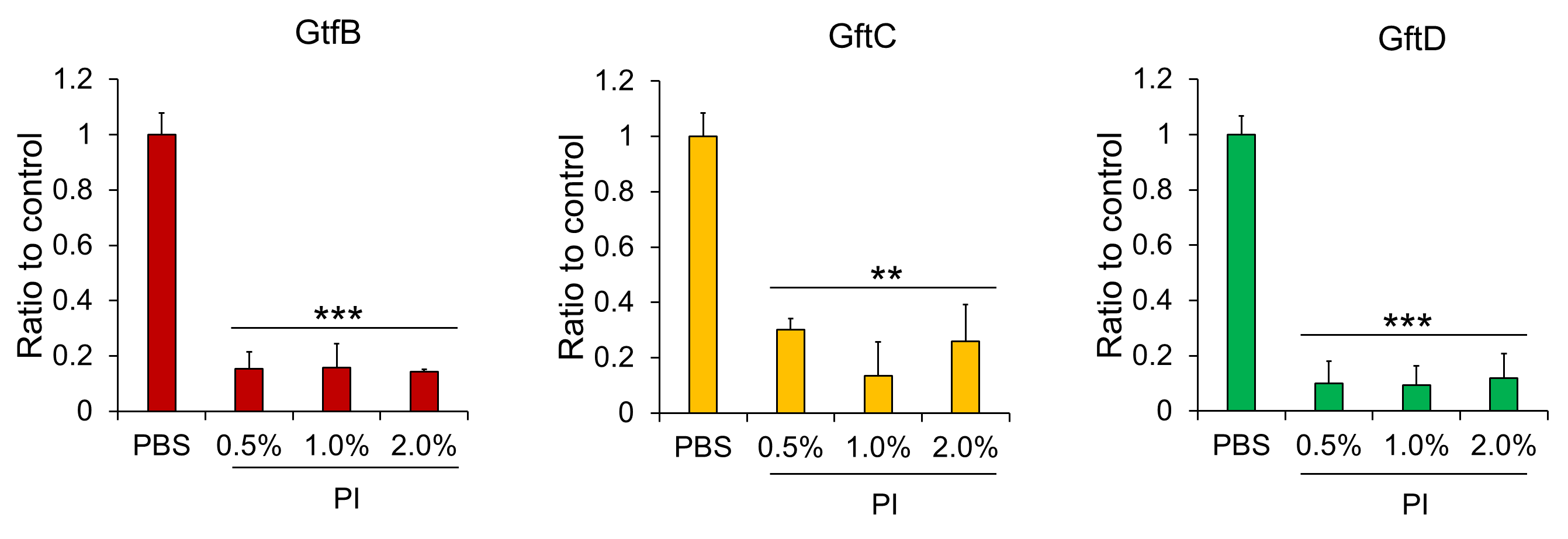
**

**Supplementary Figure 3. Effect of PI on Gtfs activity in solution.** The PI (final concentration ranged from 0.5 to 2%) significantly inhibited the enzymatic activity of all Gtfs (GtfB, red bar; GtfC, yellow bar; GtfD, green bar). Data represent mean ± s.d. (n = 3). The quantitative data were subjected to analysis of variance (ANOVA) in the Tukey’s HSD test for a multiple comparison. Values are significantly different from each other at ** *P*<0.01, ****P*<0.001 (vs. PBS control).


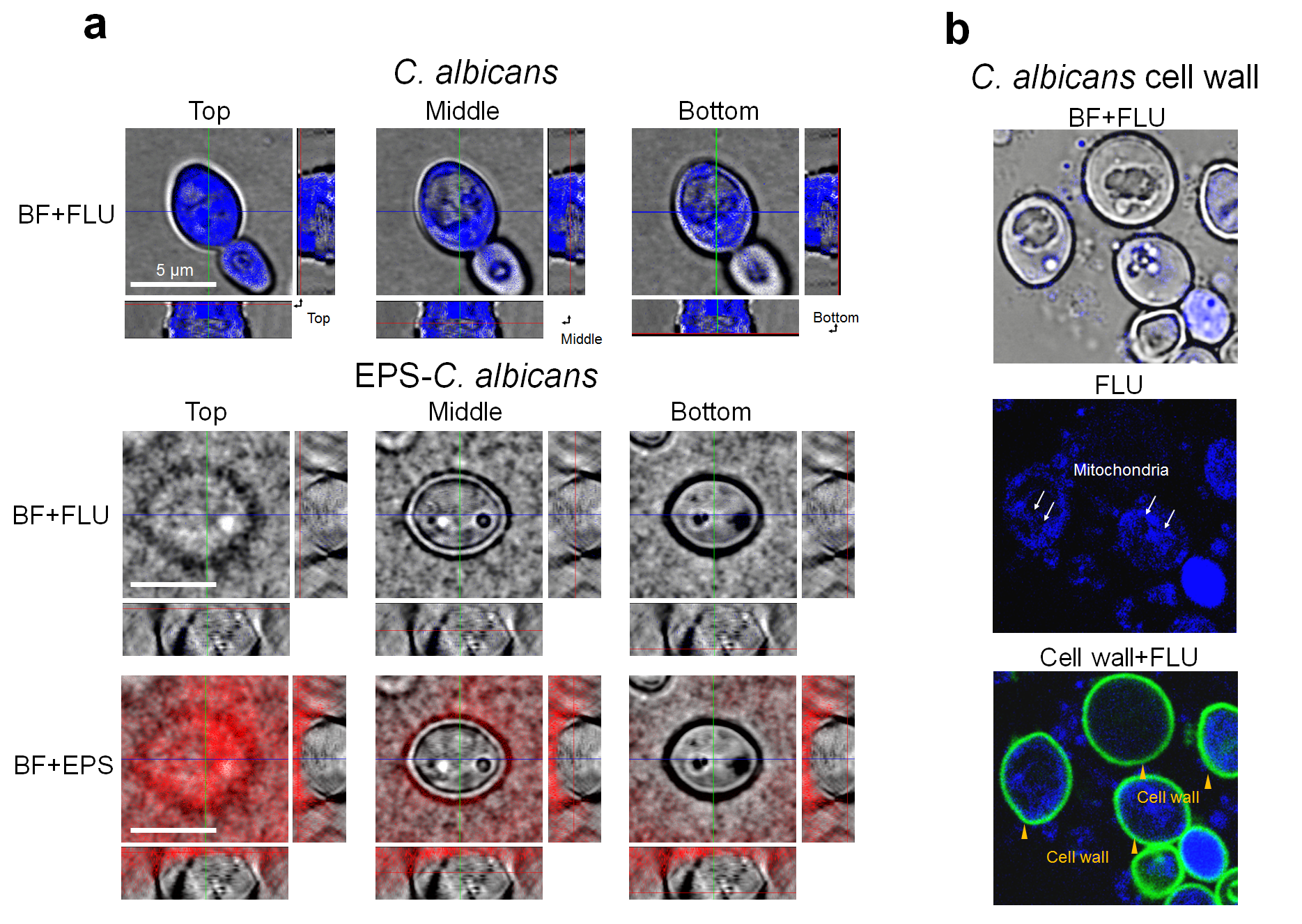


**Supplementary Figure 4. High magnification images for visualization of intracellular uptake and subcellular co-localization of fluconazole (FLU).** Detailed three-dimensional imaging analysis showing projection (top view) and orthogonal (side) views (**a**). Top panels show fluconazole (depicted in blue) fully transported and localized inside the uncoated *C. albicans*. In contrast, no fluorescent signal was detected intracellularly across the single-cell structure of EPS-enmeshed *C. albicans* (EPS is depicted in red). Co-localization of fluconazole across *Candida* cell (**b**). Counterstaining with fluorescent dye-conjugated lectin concanavalin A (that specifically the binds to cell wall) indicated that FLU is localized mostly inside the cell, but rarely found in the cell wall.

^
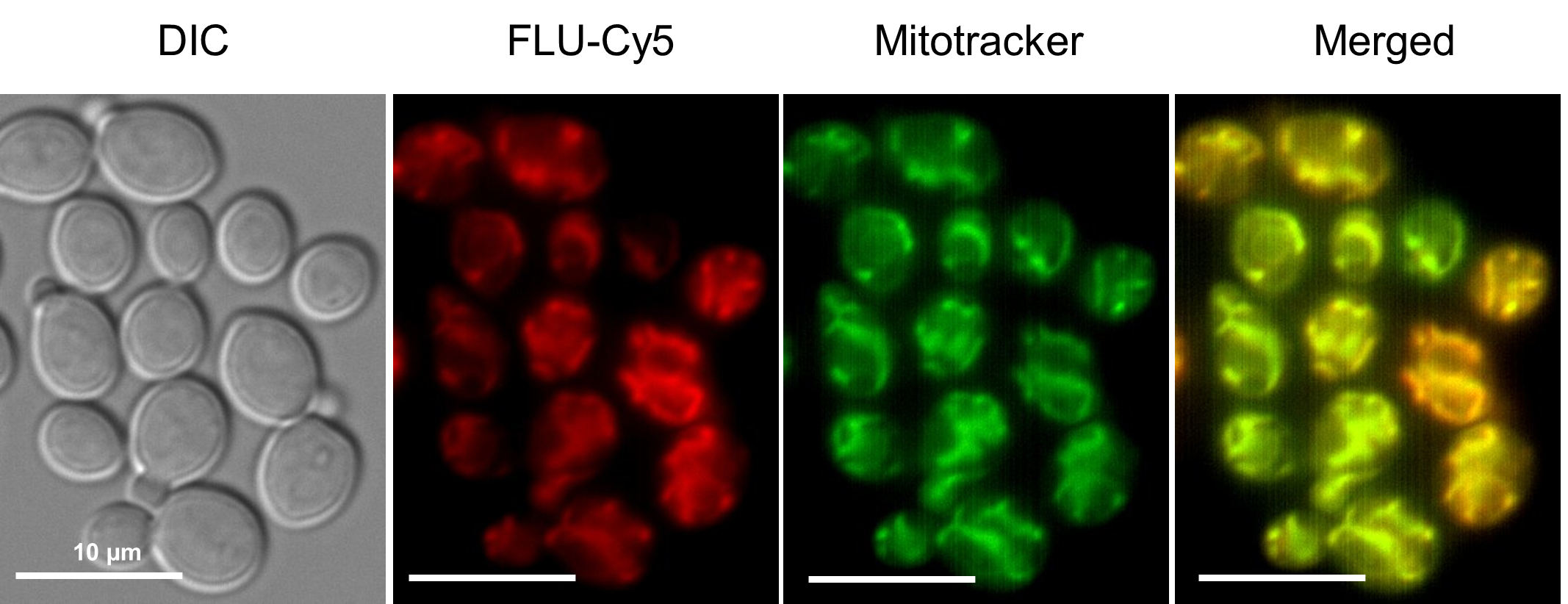
^

**Supplementary Figure 5. Localization of fluconazole (FLU)-Cy5 dye with the mitochondria.** Fluorescence imaging using Mitotracker Green FM stain (green), a mitochondria-specific labeling fluorophore (Thermo Fisher Scientific, Walthom, MA, USA), revealed that fluconazole (red) was co-localized with mitochondria (Benhamou et al., 2017). DIC, differential interference contrast.

Reference: Benhamou RI, Bibi M, Steinbuch KB, Engel H, Levin M, Roichman Y, et al. (2017). Real-time imaging of the azole class of antifungal drugs in live Candida cells. *ACS Chem Biol* **12**: 1769–1777.

^
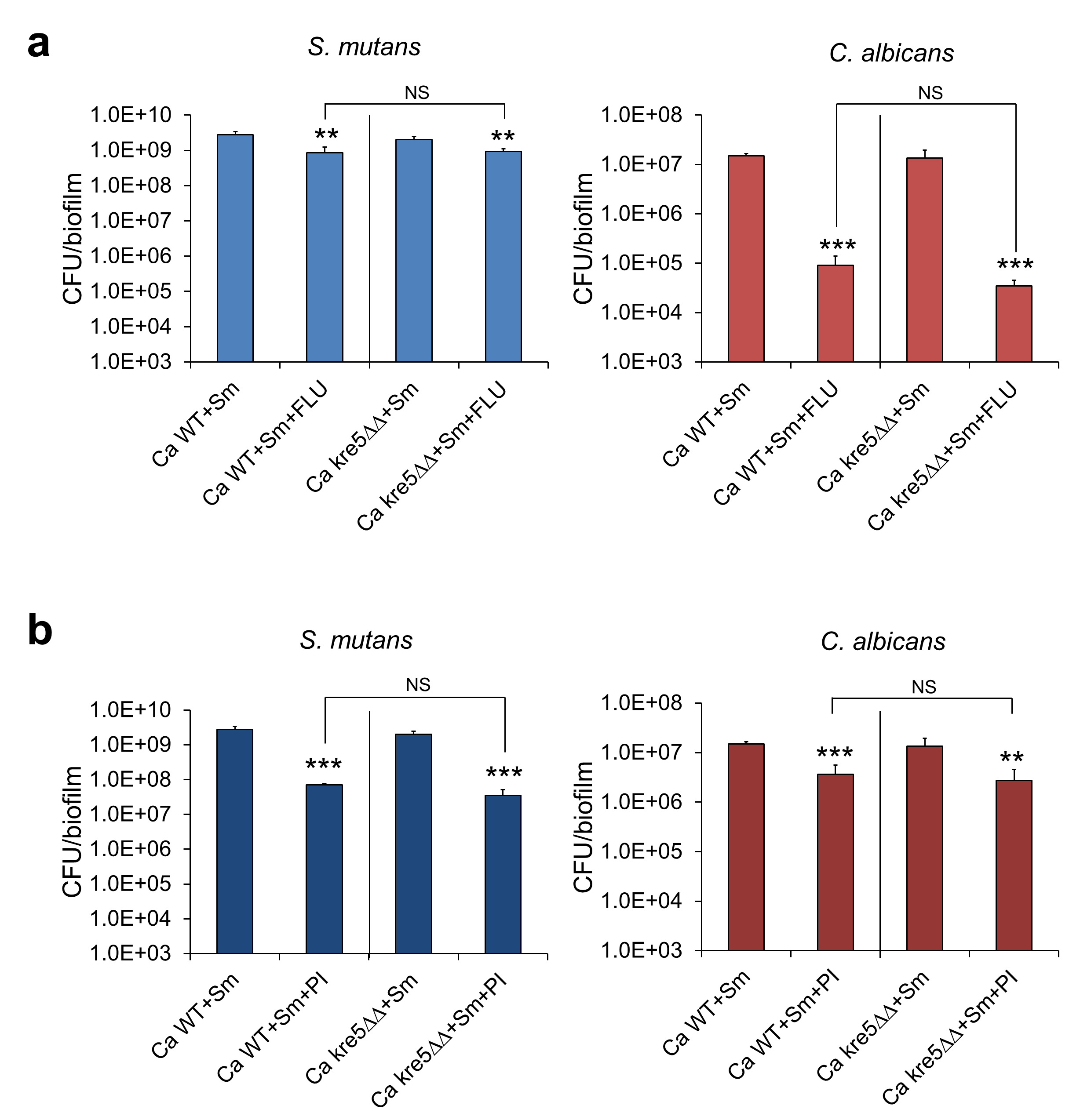
^

**Supplementary Figure 6. Influence of topical treatment of fluconazole (FLU) and povidone iodine (PI) on mixed-species biofilms co-cultured with matrix-defective *C. albicans* (*kre5∆∆*).**  CFU recovery from biofilms following treatment of FLU (**a**) and PI (**b**) (n = 4). In FLU treated biofilms, the data show comparable counts of viable fungal cells between *C. albicans* WT (SN152) and *kre5∆∆* when co-cultured with *S. mutans* (*P*>0.05), consistent with the protective role provided by *S. mutans*-derived EPS-matrix (as shown in Fig. 6). As expected, no drastic changes of *S. mutans* cell viability were observed following FLU treatment. In addition, PI had a similar antibacterial activity against *S. mutans* in mixed biofilm with either *C. albicans* SN152 or *C. albicans* *kre5∆∆* (*P*>0.05). Data represent mean ± s.d (n = 4). Values between treated and untreated biofilms (for either Ca WT or Ca *kre5∆∆*) are significantly different from each other at ***P*<0.01, ****P*<0.001 by two-tailed *t*-test. NS, non-significant.

**
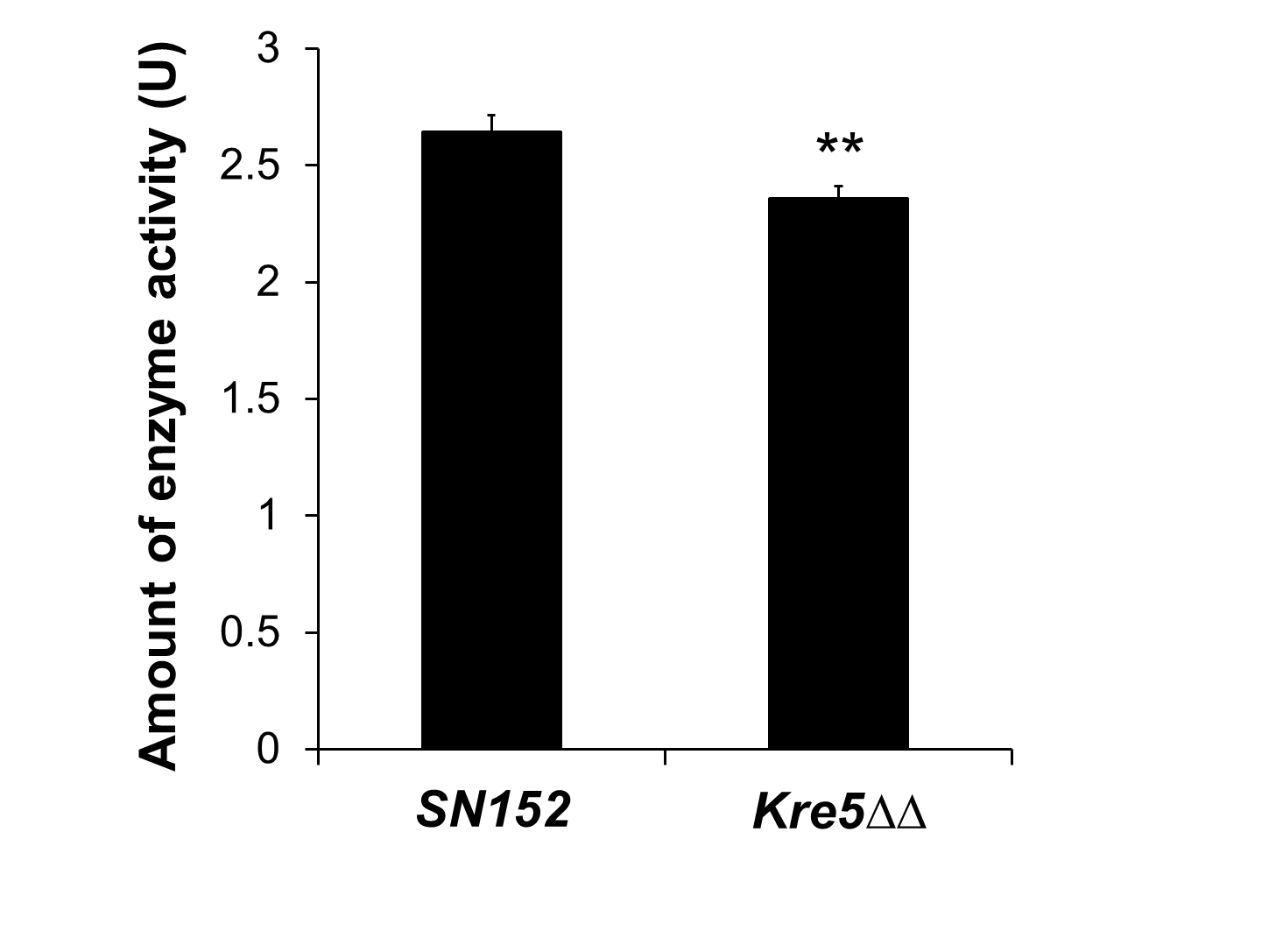
**

**Supplementary Figure 7. Determination of GtfB binding-activity onto *C. albicans* cell surface.** *C. albicans* SN152 or *C. albicans* *kre5∆∆* cells were incubated with GtfB and washed three-times to remove unbound enzyme. *Candida* cells (with or without bound GtfB) were then exposed to [^14^C-glucose]-sucrose and glucan formation was measured using scintillation counting. One unit of GtfB activity was defined as the amount of enzyme that incorporated 1 μM of glucose into glucan over 4 h. Data represent mean ± s.d (n = 3). Values are significantly different from each other at ***P*<0.01 by two-tailed *t*-test (vs*. C. albicans* SN152).


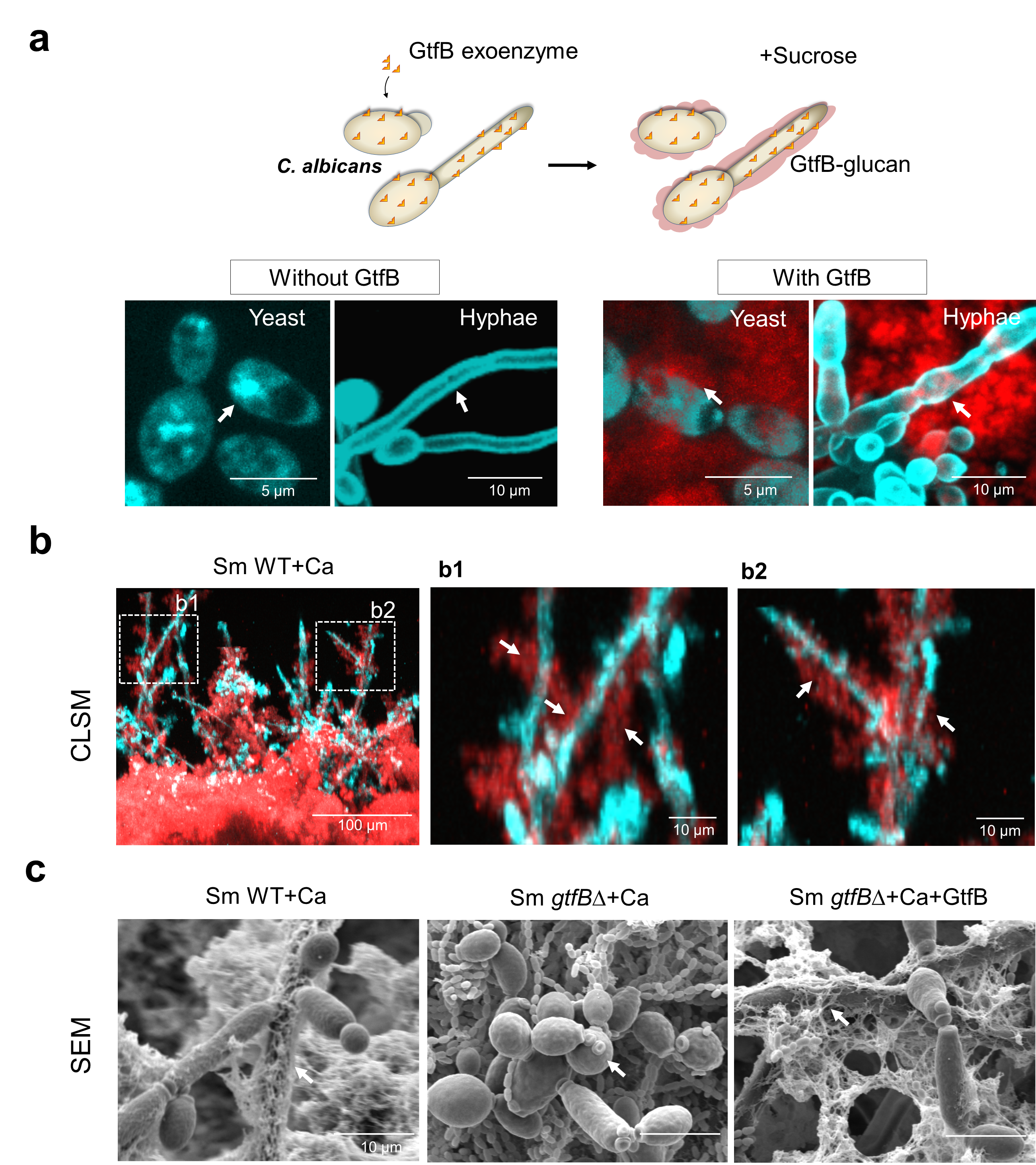


**Supplementary Figure 8. *In situ* glucan formation by GtfB bound onto *C. albicans* cell surface.** Schematics illustrating EPS glucan formation by GtfB bound onto fungal surface. Bacterially derived GtfB binds to *C. albicans* cell surfaces in active form and produces glucans (shown in red) on the fungal surface in the presence of sucrose (**a**). Mixed-species biofilm showing EPS production on *Candida* cell surface. Fungal cells are labeled with concanavalin A-tetramethylrhodamine (blue) while EPS glucans were labeled with Alexa Fluor 647 (red). Close-up images show localized EPS glucan accumulation on *C. albicans* hyphae (**b**). The presence of GtfB is critical for the EPS glucan formation on the fungal surface (**c**). SEM of *S. mutans* wild type with *C. albicans* (Sm WT+Ca), *S. mutans* *gtfB*-defective mutant with *C. albicans* (Sm *gtfB*Δ+Ca) and *S. mutans* *gtfB*Δ with *C. albicans* supplemented with purified GtfB (Sm *gtfB*Δ+Ca+GtfB). Arrow in the middle panel indicates absence of EPS matrix on the *Candida* surface, while the arrow in the right panel shows abundant EPS around fungal cells when supplemented with GtfB.
