# Supplementary material for: Bacterial-derived exopolysaccharides enhance antifungal drug tolerance in a cross-kingdom oral biofilm
Source: ISME J. 2018 Apr 18;12(6):1427–42. doi: 10.1038/s41396-018-0113-1 (PMC5955968; doi:10.1038/s41396-018-0113-1)
Supplement: Supplementary file 2 — Supplementary Materials and Methods [file 41396_2018_113_MOESM2_ESM.docx]

**Supplementary Materials and Methods**

**Bacterial-derived exopolysaccharides enhance antifungal drug tolerance in a cross-kingdom oral biofilm**

Dongyeop Kim^a^, Yuan Liu^a^, Raphael I. Benhamou^b^, Hiram Sanchez^c^, Áurea Simón-Soro^a^, Yong Li^a^, Geelsu Hwang^a^, Micha Fridman^b^, David R. Andes^c^, Hyun Koo^a^

^a^ Biofilm Research Laboratory, Department of Orthodontics and Divisions of Pediatric Dentistry & Community Oral Health, School of Dental Medicine, University of Pennsylvania, Philadelphia, PA, USA

^b^ School of Chemistry, Raymond & Beverly Sackler Faculty of Exact Sciences, Tel Aviv University, Tel Aviv, Israel

^c^ Departments of Medicine and Medical Microbiology and Immunology, University of Wisconsin, Madison, WI, USA

**Materials and methods**

**DNA extraction and PCR amplification.** Cells were pelleted from plaque-biofilms, by centrifuging at maximum speed for 5 minutes. DNA was extracted from the pellets using the Qiagen DNeasy PowerSoil HTP kit (Qiagen, Valencia, CA, USA) according to the manufacturer’s instructions. Mock washes and mock extractions were included to control for microbial DNA contamination arising through the sonication and extraction processes, respectively. PCR amplification of V1-V2 region of 16S rRNA gene was performed using Golay-barcoded universal primers 27F and 338R (Kim et al., 2017). Four replicate PCR reactions were performed for each sample using Q5 Hot Start High Fidelity DNA Polymerase (New England BioLabs, Ipswich, MA, USA). Each PCR reaction contained: 4.3 μl microbial DNA-free water, 5 μl 5× buffer, 0.5 μl dNTPs (10 mM), 0.17 μl Q5 Hot Start Polymerase, 6.25 μl each primer (2 μM), and 2.5 μl DNA. PCR reactions with no added template or synthetic DNAs were performed as negative and positive controls, respectively. PCR amplification was done on a Mastercycler Nexus Gradient thermal cycler (Eppendorf, Hamburg, Germany) using the following conditions: DNA denaturation at 98°C for 1 min, then 20 cycles of denaturation 98°C for 10 s, annealing 56°C for 20 s and extension 72°C for 20 s, last extension was at 72°C for 8 min. PCR replicates were pooled and then purified using a 1:1 ratio of Agencourt AMPure XP beads (Beckman Coulter, Indianapolis, IN, USA), following the manufacturer’s protocol. The final library was prepared by pooling 10 μg of amplified DNA per sample. Those that did not arrive at the DNA concentration threshold (e.g. negative control samples) were incorporated to the final pool by adding 12 μl. The library was sequenced to obtain 2×250 bp paired-end reads using the MiSeq Illumina (Illumina, San Diego, CA, USA) (Caporaso et al., 2012).

**Quantitative biofilm analysis.** The biofilms formed in each condition were examined using confocal laser scanning microcopy (CLSM) combined with quantitative computational analysis and microbiological assays. Briefly, *S. mutans* were stained with 2.5 µM SYTO 9 green-fluorescent nucleic acid stain (485/498 nm; Molecular Probes Inc., Eugene, OR, USA) and *C. albicans* cells were stained with concanavalin A (ConA) lectin conjugated with tetramethylrhodamine at 40 μg/ml (555/580 nm; Molecular Probes, Inc.), while EPS was labelled with 1 µM Alexa Fluor 647-dextran conjugate (647/668 nm; Molecular Probes Inc.) as detailed previously (Xiao et al., 2012; Falsetta et al., 2014). The confocal images of biofilms were obtained with a single-photon confocal microscope (LSM800, Zeiss, Jena, Germany) and a multi-photon laser scanning microscope (SP8, Leica Microsystems, Buffalo Grove, IL, USA) equipped with a 20 × (1.0 numerical aperture) water immersion lens. The biofilm were excited at 840 nm, and the fluorescence emitted was collected with the hybrid photodetectors (SYTO 9, 490–540 nm; ConA, 550–628 nm; Alexa Fluor 647, 645–750 nm). The confocal images were analyzed using COMSTAT to calculate the EPS-biovolume (Xiao et al., 2012). Amira 5.4.1 software (Visage Imaging, San Diego, CA, USA) was used to create 3D renderings to visualize the overall architecture of the biofilms. Furthermore, a separate set of biofilms was used for standard microbiological analysis. The biofilms were homogenized by sonication, and the number of viable cells (total number of colony forming units (CFU) per biofilm) was determined as described elsewhere (Koo et al., 2003). Biochemical quantification of water-insoluble EPS was performed via an established colorimetric (phenol-sulfuric acid method) assay (Koo et al., 2003).

***In vivo* rodent animal model.** Animal experiments were performed on a well-established rodent model as described elsewhere (Falsetta et al., 2014; Hwang et al., 2017). Briefly, 15 days-old female Sprague-Dawley rat pups were purchased with their dams from Envigo (Madison, WI, USA). Upon arrival, animals were screened for *S. mutans* and *C. albicans,* and were determined not to be infected with either organism, by plating oral swabs on selective media: Mitis Salivarius agar plus bacitracin (MSB) for *S. mutans* and Sabouraud dextrose broth (SAB) agar containing chloramphenicol or ChromAgar for *C. albicans*. The animals were then infected by mouth with actively growing (mid-logarithmic) culture of *S. mutans* UA159 and *C. albicans* between 19 and 25 days, and their infection with both organisms confirmed at 26 days as detailed previously (Falsetta et al., 2014). All the animals were randomly placed into treatment groups, and their teeth were treated topically twice daily with brief 30 s-exposure (to simulate the clinical situation) using a custom-made applicator. The treatment groups included: (1) control (PBS), (2) povidone iodine (PI, 2% vol/vol), (3) fluconazole (FLU, 0.2% wt/vol (2 mg/ml)), and (4) PI+FLU. Each group was provided the National Institutes of Health cariogenic diet 2000 and 5% sucrose water *ad libitum*. The experiment proceeded for 3 week (21 days). At the end of the experimental period, the animals were sacrificed, and the jaws were surgically removed and aseptically dissected; the left jaws were sonicated in sterile saline solution and co-infection of *S. mutans* and *C. albicans* in all groups were confirmed as determined using a plating (selective media) and qPCR methods using species-specific primers as described previously (Hwang et al., 2017). The right jaws were directly fixed and the morphology and colonization of co-species biofilm were characterized using a high-resolution environmental scanning electron microscopy (Quanta 250 FEG eSEM, FEI, Hillsboro, OR, USA) at Electron Microscopy Resource Lab, University of Pennsylvania.

**Glucosyltransferases enzymatic activity.** The influence of PI on the activities of GtfB, GtfC and GtfD in solution phase was determined as described previously (Koo et al., 2002). Briefly, Gtfs in the absorption buffer (pH 6.5) were mixed with PI (at concentrations ranging from 0.5% to 2%) or PBS control, and then incubated with a [^14^C-glucose]-sucrose substrate (0.2 µCi/ml; 200 mM of sucrose, 40 µM dextran 900, and 0.02% NaN_3_ in buffer consisting of 50 mM KCl, 1 mM CaCl_2_, and 0.1 mM MgCl_2_ at pH 6.5) at 37^o^C for 4 h. One unit of GtfB activity was defined as the amount of enzyme that incorporated 1 μM of glucose into glucan over the reaction time as determined via scintillation counting.

**GtfB binding to the *C. albicans* cell surface.** *C. albicans* SN152 or *C. albicans* *kre5∆∆* cells were incubated with GtfB and washed three-times to remove unbound enzyme. *Candida* cells (with or without bound GtfB) were then exposed to [^14^C-glucose]-sucrose and glucan formation was measured using scintillation counting as detailed by Gregoire et al. (2011).

**Sequestration of ^3^H-fluconazole in EPS-enmeshed *C. albicans*.** Radiolabeled fluconazole (^3^H, Moravek Biochemicals, Brea, CA, USA) was used to assess drug retention within EPS-matrix surrounding *C. albicans* cells. After enmeshing the cells in EPS-matrix via *in situ* production of α-glucans (as described above), they were incubated with 7×10^5^ CPM of ^3^H-fluconazole (final conc. 0.5 μM) in UFTYE medium at 37°C for 1 h. Unlabeled fluconazole (20 μM) in UFTYE medium was added for additional 15 min incubation. After centrifugation (at 3400×g, 4°C for 20 min), cells were collected and an aliquot of each collected intact cell was saved for scintillation counting as described elsewhere (Mitchell et al., 2015). The EPS-enmeshed cells were disrupted by vortexing and sonication to separate cell and matrix. Extracellular matrix was further extracted and fractionated using 1N NaOH at 37°C for 1 h. Cells were subsequently disrupted by bead beating, and the intracellular and cell wall portions were collected by centrifugation. The values of ^3^H-fluconazole sequestered in EPS-*C. albicans* were compared with bare *C. albicans* and expressed as relative ratio.

**References**

Caporaso JG, Lauber CL, Walters WA, Berg-Lyons D, Huntley J, Fierer N et al. (2012). Ultra-high-throughput microbial community analysis on the Illumina HiSeq and MiSeq platforms. *ISME J* **6**:1621-1624.

Falsetta ML, Klein MI, Colonne PM, Scott-Anne K, Gregoire S, Pai CH et al. (2014). Symbiotic relationship between *Streptococcus mutans* and *Candida albicans* synergizes virulence of plaque biofilms in vivo. *Infect Immun* **82**: 1968–1981.

Gregoire S, Xiao J, Silva BB, Gonzalez I, Agidi PS, Klein MI, et al. (2011). Role of glucosyltransferase B in interactions of *Candida albicans* with *Streptococcus mutans* and with an experimental pellicle formed on hydroxyapatite surfaces. *Appl Environ Microbiol* **77**: 6357–6367.

Hwang G, Liu Y, Kim D, Li Y, Krysan DJ, Koo H. (2017). *Candida albicans* mannans mediate *Streptococcus mutans* exenyzme GtfB binding to modulate cross-kingdom biofilm development *in vivo*. *PLoS Pathog* **13**: e1006407.

Kim D, Hofstaedter CE, Zhao C, Mattei L, Tanes C, Clarke E et al. (2017). Optimizing methods and dodging pitfalls in microbiome research. *Microbiome* **5**:52.

Koo H, Hayacibara MF, Schobel BD, Cury JA, Rosalen PL, Park YK et al. (2003). Inhibition of *Streptococcus mutans* biofilm accumulation and polysaccharide production by apigenin and *tt*-farnesol. *J Antimicrob Chemother* **52**: 782–789.

Koo H, Rosalen PL, Cury JA, Park YK, Bowen WH. (2002). Effects of compounds found in propolis on *Streptococcus mutans* growth and on glucosyltransferase activity. *Antimicrob Agents Chemother* **46**: 1302–1309.

Mitchell KF, Zarnowski R, Sanchez H, Edward JA, Reinicke EL, Nett JE et al. (2015). Community participation in biofilm matrix assembly and function. *Proc Natl Acad Sci USA* **112**: 4092–4097.

Xiao J, Klein MI, Falsetta ML, Lu B, Delahunty CM, Yates JR, et al. (2012). The exopolysaccharide matrix modulates the interaction between 3D architecture and virulence of a mixed-species oral biofilm. *PLoS Pathog* **8**: e1002623.
